# Cell cycle arrest determines adult neural stem cell ontogeny by an embryonic Notch-nonoscillatory *Hey1* module

**DOI:** 10.1038/s41467-021-26605-0

**Published:** 2021-11-12

**Authors:** Yujin Harada, Mayumi Yamada, Itaru Imayoshi, Ryoichiro Kageyama, Yutaka Suzuki, Takaaki Kuniya, Shohei Furutachi, Daichi Kawaguchi, Yukiko Gotoh

**Affiliations:** 1 Graduate School of Pharmaceutical Sciences, The University of Tokyo, Tokyo, 113-0033 Japan; 2 Institute for Frontier Life and Medical Sciences, Kyoto University, Kyoto, 606-8507 Japan; 3 Research Center for Dynamic Living Systems, Graduate School of Biostudies, Kyoto University, Kyoto, 606-8501 Japan; 4 Department of Computational Biology and Medical Sciences, Graduate School of Frontier Sciences, The University of Tokyo, Chiba, 277-8562 Japan; 5 International Research Center for Neurointelligence (WPI-IRCN), The University of Tokyo, Tokyo, 113-0033 Japan; 6 Present Address: RIKEN Center for Brain Science, 2-1 Hirosawa, Wako, Saitama, 351-0198 Japan; 7 Present Address: Sainsbury Wellcome Centre, University College London, 25 Howland Street, London, W1T 4JG UK

**Keywords:** Neurogenesis, Neural stem cells, Cell fate and cell lineage, Neural progenitors, Quiescence

## Abstract

Quiescent neural stem cells (NSCs) in the adult mouse brain are the source of neurogenesis that regulates innate and adaptive behaviors. Adult NSCs in the subventricular zone are derived from a subpopulation of embryonic neural stem-progenitor cells (NPCs) that is characterized by a slower cell cycle relative to the more abundant rapid cycling NPCs that build the brain. Yet, how slow cell cycle can cause the establishment of adult NSCs remains largely unknown. Here, we demonstrate that Notch and an effector Hey1 form a module that is upregulated by cell cycle arrest in slowly dividing NPCs. In contrast to the oscillatory expression of the Notch effectors Hes1 and Hes5 in fast cycling progenitors, Hey1 displays a non-oscillatory stationary expression pattern and contributes to the long-term maintenance of NSCs. These findings reveal a novel division of labor in Notch effectors where cell cycle rate biases effector selection and cell fate.

## Abstract (web summary)

Adult neural stem cells are derived from an embryonic population of slowcycling progenitor cells, though how reduced cycling speed leads to establishment of the adult population has remained elusive. Here they show that non-oscillatory Notch-Hey signaling induced by slow-cycling contributes to long term maintenance of neural stem cells.

## Introduction

In many adult mammalian tissues, somatic stem cells are maintained in a quiescent state and are available to generate differentiated cells on demand^1^. Quiescence is necessary to maintain these adult tissue stem cells over the long term^2–9^. Adult neural stem cells (NSCs) in the mouse subventricular zone (SVZ) are such stem cells that are preserved in a quiescent state^10,11^. Once activated, NSCs undergo proliferation and differentiation, producing interneurons that migrate into the olfactory bulb^10,12,13^, where they contribute to the rewiring of existing neuronal circuitry, and associated cognitive functions and innate behaviors^13,14^. Recent studies have revealed the lineage relation between embryonic neural stem-progenitor cells (NPCs, also known as radial glial cells) and adult NSCs in this neurogenic niche. Most adult NSCs in the lateral wall of the SVZ are derived from a quiescent or slowly dividing subpopulation of embryonic NPCs, while the rapidly dividing subpopulation give rise to neurons and glial cells that populate the brain during development^15,16^. High expression of the cyclin-dependent kinase inhibitor (CKI) p57 in the slowly dividing subpopulation inhibits proliferation and neural differentiation and is required for genesis of adult NSCs^16^. However, the molecular and cellular mechanisms by which embryonic cell cycle inhibition maintains the undifferentiated state of embryonic NPCs resulting in the emergence of a stable population of postnatal adult NSCs remains unknown.

Notch signaling plays a central role in maintenance of the undifferentiated state of both rapidly dividing embryonic NPCs and adult NSCs in the mammalian central nervous system^17–22^, but its role in the slowly dividing embryonic precursors of adult NSCs is unknown. The mammalian Notch proteins (Notch1–4) are transmembrane receptors that are activated by the ligands Delta-like and Jagged, triggering cleavage of the Notch intracellular domain (NICD), which translocates to the nucleus and induces the expression of Notch effector genes including those for the Hes/Hey family of transcriptional repressors e.g., Hes1, Hes5, Hey1 (Hesr1), Hey2 (Hesr2), and HeyL^23–27^. In turn, the expression of Hes/Hey family proteins results in the suppression of proneural genes such as those for Ascl1, Neurog1, and Neurog2, thereby inhibiting neuronal differentiation, and maintaining the undifferentiated state of NPCs^28–35^. In the Hes/Hey family, Hes1 and Hes5 are mainly responsible for mediating Notch signaling that underlies the maintenance of rapidly dividing NPCs in the neocortex^36–40^. Hes1 and Hes5 show oscillatory expression dynamics as a result of negative feedback where Hes proteins suppress their own gene promoters, and due to the short half-lives of their mRNAs and proteins^41,42^. The oscillatory expression dynamics of Hes1 and Hes5 in turn result in the oscillatory expression of their target genes such as *Ascl1*, *Neurog2*, and *Olig2*, enabling embryonic NPCs to undergo frequent proliferation and differentiation^31,42,43^. Much less is known, however, on whether and how Notch signaling is differentially regulated in the subpopulation of slowly dividing embryonic NPCs that gives rise to adult NSCs. Mechanistically, it remains to be determined how cell cycle arrest is causally linked to the unique features of the adult NSC lineage such as long-term maintenance and stability.

Here, in the developing mouse lateral ganglionic eminence (LGE), we show that active Notch1 and Hey1 are more abundant in slowly dividing NPCs than in their rapidly dividing counterparts. We demonstrate that Hey1 is necessary for the robust maintenance of slowly dividing NPCs that persist to postnatal stages but dispensable for the rapidly dividing NPCs. The *Hey1* promoter drives non-oscillatory expression dynamics that are qualitatively different from the notable oscillatory expression conferred by the *Hes5* promoter. Our results suggest that the persistent and high level of Hey1 expression in NPCs is responsible for robust maintenance of the undifferentiated state in slowly dividing NPCs from the embryonic to postnatal stages, and that it serves as the earliest known regulator of the adult NSC lineage.

## Results

### Embryonic NPC cell cycle inhibition shifts the transcriptional profile toward that of adult quiescent NSCs

**Fig. 1:**
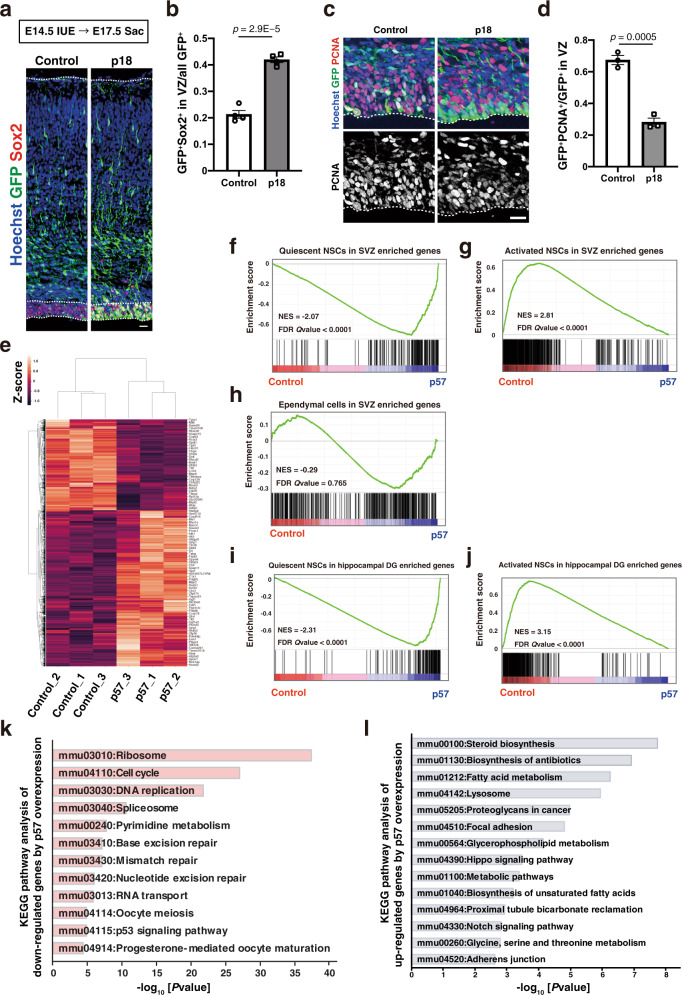
p57 overexpression shifts the global transcriptional profile close to that of adult quiescent NSCs. **a**, **c** Immunohistochemical analysis with antibodies to GFP and to either Sox2 (**a**) or PCNA (**c**) for brain sections prepared at E17.5 after in utero electroporation (IUE) with an expression plasmid encoding mouse p18 and GFP or with the corresponding plasmid encoding GFP alone (control) at E14.5. Nuclei were stained with Hoechst 33342. Scale bars, 20 μm. Dashed lines indicate the ventricular zone and the pial surface. **b** Quantification of the proportion of GFP^+^Sox2^+^ cells among all GFP^+^ cells. Five brain sections were analyzed per embryo. Data are means ± SEM (*n* = 4 embryos), two-tailed Student’s *t* test. **d** Quantification of the proportion of GFP^+^PCNA^+^ cells among all GFP^+^ cells in the VZ. Five brain sections were analyzed per embryo. Data are means ± SEM (*n* = 3 embryos), two-tailed Student’s *t* test. **e** Hierarchical clustering of the 3920 differentially expressed genes (DEGs, edgeR; *p* < 0.05). **f**–**h** GSEA of the adult quiescent NSCs (**f**), activated NSCs (**g**), and ependymal cells (**h**) in SVZ signature genes^46^ in control embryonic NPCs versus p57-expressing embryonic NPCs. NES normalized enrichment score; FDR false discovery rate. **i**, **j** GSEA of the adult quiescent NSCs (**i**) and activated NSCs (**j**) in hippocampal dentate gyrus signature genes^47^ in control embryonic NPCs versus p57-expressing embryonic NPCs. **k** KEGG pathway analysis of control NPCs enriched genes (edgeR; *p* < 0.03, expression top 800). **l** KEGG pathway analysis of p57-expressing NPCs enriched genes (edgeR; *p* < 0.03, expression top 1000).

We previously demonstrated that forced expression of p57, which induces cell cycle arrest at either G_1_ or G_2_ phase, in rapidly dividing NPCs of the developing mouse neocortex suppresses neuronal differentiation and increases the fraction of NPCs positive for the undifferentiated state marker Sox2 in a manner dependent on its CKI domain^16^ (Supplementary Fig. 1a, b). We found that forced expression of p18, which belongs to another branch of the CKI family and induces G_1_ arrest, in neocortical NPCs at embryonic day (E) 14.5 also resulted in an increase in the Sox2^+^ fraction and a decrease in the fraction of cells positive for proliferating cell nuclear antigen (PCNA) in the ventricular zone (VZ) at E17.5 (Fig. 1a–d). Given that the CKI domains of p57 and of p18 belong to distinct classes that evolved separately and have distinct binding partner specificities^44^, these results indicate that cell cycle inhibition (presumably at G_1_ phase) promotes maintenance of the undifferentiated state of NPCs.

We next investigated whether cell cycle inhibition might be sufficient to reprogram embryonic NPCs towards adult NSCs in addition to maintaining the undifferentiated state. We thus used RNA sequencing (RNA-Seq) to analyze the effect of p57 overexpression on the transcriptional profile of embryonic neocortical NPCs at E17.5 (Fig. 1e–l and Supplementary Fig. 1c–i). An unsupervised cluster analysis segregated p57-expressing embryonic NPCs from control embryonic NPCs and revealed prominent differences in their transcriptional profiles, with 3809 differentially expressed genes (DEGs) (Fig. 1e and Supplementary Fig. 1d). We then compared these DEGs with those between quiescent and activated NSCs in the adult SVZ^45,46^. A gene set enrichment analysis (GSEA) showed a remarkable enrichment of quiescent NSC signature genes in p57-expressing embryonic NPCs compared to control embryonic NPCs (Fig. 1f and Supplementary Fig. 1e). On the other hand, activated NSC signature genes were significantly enriched in control embryonic NPCs (Fig. 1g and Supplementary Fig. 1f). These trends were also the case when we used the data of quiescent and activated NSCs in the hippocampal dentate gyrus^47^ (Fig. 1i, j). Gene ontology (GO) and pathway analyses of DEGs showed that downregulated genes in embryonic NPCs by p57 expression include those related to the cell cycle (Fig. 1k and Supplementary Fig. 1h). However, the striking enrichment shown in Fig. 1f was not only due to the cell cycle inhibition, given that signature genes of adult ependymal cells, which are also known to be derived from quiescent embryonic NPCs^48^ did not show significant enrichment in p57-expressing embryonic NPCs (Fig. 1h). Instead, GO and pathway analyses of the DEGs upregulated by p57 expression in embryonic NPCs include those related to lipid metabolism, steroid biosynthesis, lysosome, and cell adhesion (Fig. 1l and Supplementary Fig. 1i), which are also enriched in signature genes of quiescent adult NSCs but not those of adult ependymal cells^46^. These results indicate that p57 expression in embryonic NPCs is sufficient to skew their expression profile close to that of quiescent adult NSCs.

### Cell cycle inhibition activates Notch signaling in embryonic neocortical NPCs

We then asked what molecular changes might promote the maintenance of NPCs in response to cell cycle inhibition described in Supplementary Fig. 1a, b. We found that p57 overexpression upregulated transcription signatures of the Notch signaling pathway (Fig. 1l and Supplementary Fig. 1i). Indeed, p57 overexpression increased the mRNA levels of Notch receptors (*Notch2* and *Notch3*), Notch downstream effectors (*Hes5*, *Hey1*, *Hey2*, and *Heyl*) and membrane proteins related to Notch activation (*S1pr3*, *Tmem100*, and *Dner*)^49–51^ (Supplementary Fig. 2a). This was surprising, given the requirement of Notch signaling even in rapidly dividing NPCs for their maintenance and division^31^. We actually noticed that NPCs overexpressing p57 or p18 contained elaborate radial fibers harboring Fabp7 (fatty acid binding protein 7), a target of Notch signaling^52^ (Supplementary Fig. 3a, b). This phenotype resembled that observed in NPCs expressing an active form of Notch1 (NICD1) (Supplementary Fig. 3c). We thus hypothesized that cell cycle inhibition in slowly cycling proto-NSCs, or induced by forced expression of p57 or p18 in NPCs, might increase the level of Notch signaling.

**Fig. 2:**
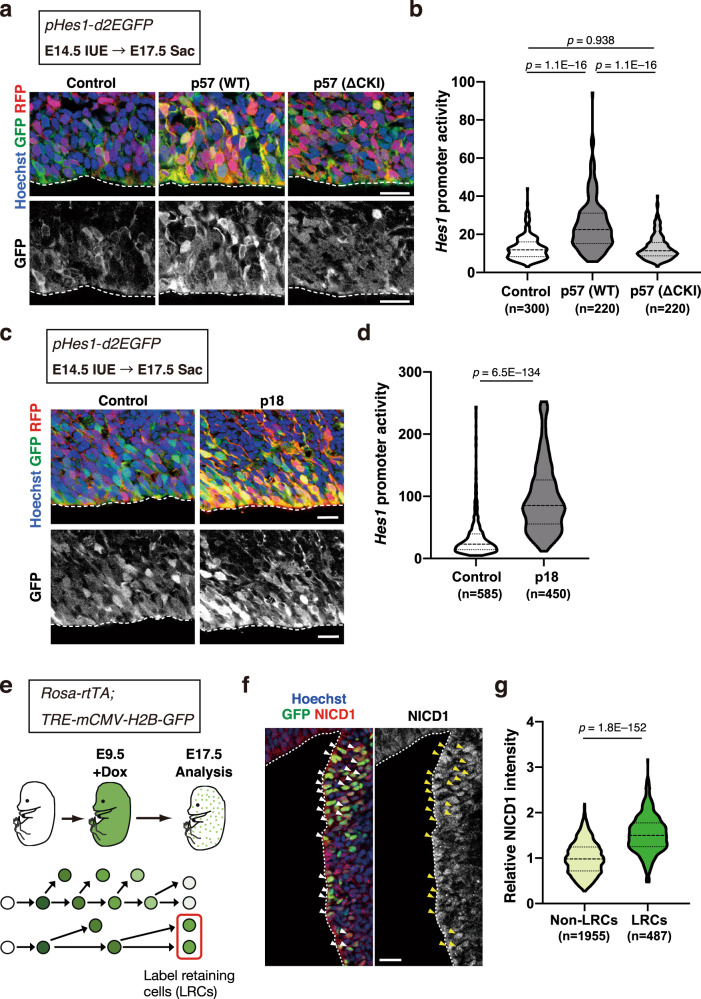
Cell cycle inhibition activates Notch signaling in embryonic NPCs. **a**, **c** Immunohistochemical analysis with antibodies to red fluorescent protein (RFP) and to GFP for detection of the d2 mutant of EGFP for sections of the *pHes1-d2EGFP* mouse neocortex prepared at E17.5 after in utero electroporation at E14.5 with an expression plasmid encoding mCherry either alone (control) or together with WT or ΔCKI mutant forms of mouse p57 (**a**) or mouse p18 (**c**). Nuclei were stained with Hoechst 33342. Scale bars, 20 μm. Dashed lines indicate the ventricular surface. **b**, **d** Quantification of GFP fluorescence intensity (reflecting the activity of the *Hes1* promoter) in the indicated numbers of RFP^+^ cells of the VZ for three independent experiments as in (**a**) and (**c**), respectively. Violin plots show the median (horizontal dashed line) and quartiles (horizontal dotted lines). *n* = 300, 220, and 220 cells for control, p57 WT, and p57 ΔCKI, respectively (**b**). *n* = 585 and 450 cells for control and p18, respectively (**d**). Five brain sections were analyzed per embryo, one-way ANOVA followed by Scheffe’s multiple comparison test (**b**) or one-sided Mann–Whitney test (**d**). **e** Scheme for labeling of slowly dividing cells on the basis of H2B-GFP retention. 9TB-Dox (0.25 mg) was injected intraperitoneally into pregnant *Rosa-rtTA;TRE-mCMV-H2B-GFP* mice at E9.5, and embryos were analyzed at E17.5. **f** Immunohistofluorescence analysis with antibodies to GFP and to NICD1 in LGE sections of *Rosa-rtTA;TRE-mCMV-H2B-GFP* embryos treated as in **e**. Nuclei were stained with Hoechst 33342. Scale bar, 20 μm. Dashed lines indicate the ventricular surface. Arrowheads indicate GFP-retaining slowly dividing cells. **g** Quantification of NICD1 staining intensity for the indicated numbers of label-retaining cells (LRCs) and non-LRCs (top 15% and bottom 60% of NPCs for H2B-GFP staining intensity, respectively) in the LGE at E17.5 for sections as in **f**. Data are expressed relative to the corresponding value for non-LRCs. Violin plots show the median (horizontal dashed line) and quartiles (horizontal dotted lines) (*n* = 1955 and 487 cells for non-LRCs and LRCs, respectively). Eight brain sections were analyzed per embryo, two-tailed Student’s *t* test.

To examine the level of Notch signaling, we monitored the activity of Notch-Hes signaling with the use of transgenic mice expressing a reporter construct (*pHes1-d2EGFP*) encoding a mutant (d2) of enhanced green fluorescent protein (EGFP) with a short half-life under the control of the mouse *Hes1* promoter. Overexpression of p57 in neocortical NPCs at E14.5 resulted in a significant increase in the reporter expression in the VZ at E17.5 (Fig. 2a, b). Moreover, the expression of a p57 mutant (ΔCKI) that lacks the CKI domain had no such effect, suggesting that p57 activates Notch signaling in a manner dependent on this domain (Fig. 2a, b). Furthermore, the overexpression of p18 significantly increased the activity of the *Hes1* promoter in this assay (Fig. 2c, d). Together, these results suggest that forced cell cycle inhibition in rapidly dividing neocortical NPCs results in the activation of the *Hes1* promoter, likely through the activation of Notch signaling.

### Notch1 is highly activated in slowly dividing embryonic NPCs

We then determined whether the activity of endogenous Notch signaling might differ between rapidly dividing and slowly dividing NPCs in the LGE with the use of an immunohistochemical analysis of NICD1. We previously showed that slowly dividing embryonic NPCs slow down their cell cycle between E13 and E15, which can be detected by an analysis of histone 2B (H2B)-GFP retention^16^. In this analysis, the expression of an H2B-GFP fusion protein was transiently induced at E9.5 by the injection of 9-*tert*-butyldoxycycline (9TB-Dox) into pregnant *Rosa-rtTA;TRE-mCMV-H2B-GFP* mice, and slowly dividing NPCs were detected as H2B-GFP–retaining NPCs^16^ (Fig. 2e). We found that the level of NICD1 was significantly higher in H2B-GFP–retaining NPCs (slowly dividing NPCs) than in non-retaining NPCs (rapidly dividing NPCs) (defined as the top 15% and bottom 60% of H2B-GFP levels, respectively, among Ascl1^–^ cells in the VZ) in the LGE at E17.5 (Fig. 2f, g). This finding indicated that Notch1 is activated to a greater extent in slowly dividing NPCs compared with rapidly dividing NPCs in the embryonic LGE.

### Hey1 is highly expressed in slowly dividing embryonic NPCs

**Fig. 3:**
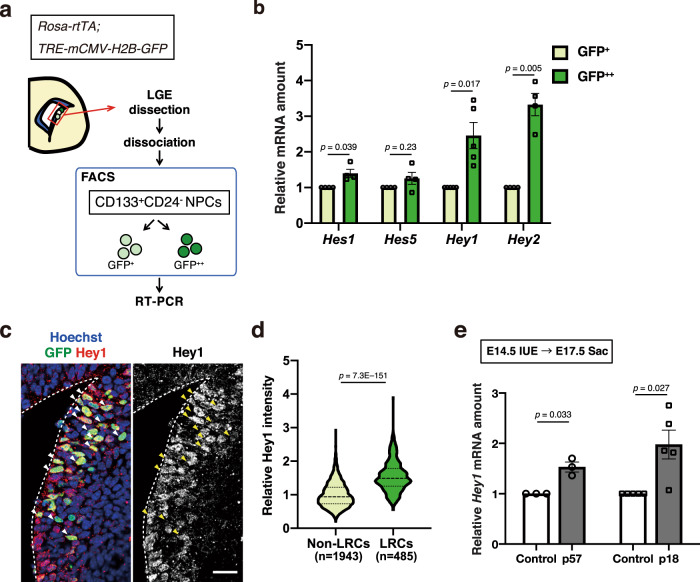
Hey1 is highly expressed in slowly dividing embryonic NPCs. **a** Scheme for the isolation of slowly dividing and rapidly dividing NPCs from the LGE of *Rosa-rtTA;TRE-mCMV-H2B-GFP* embryos at E16.5 on the basis of H2B-GFP retention after exposure to 9TB-Dox at E9.5. Embryonic NPCs were defined as CD133^+^CD24^–^ cells and were subdivided into two populations based on H2B-GFP intensity—top 8% (GFP^++^) and middle 40% to 65% (GFP^+^)—by FACS for RT and real-time PCR analysis. **b** RT and real-time PCR analysis of the indicated Notch target genes in NPCs isolated as in **a**. Data were normalized by the amount of β-actin mRNA, are expressed relative to the corresponding value for GFP^+^ cells, and are means ± SEM (*n* = 4, 4, 5, and 4 independent experiments for *Hes1*, *Hes5*, *Hey1*, and *Hey2*, respectively), two-tailed paired *t* test. **c** Immunohistochemical analysis with antibodies to GFP and to Hey1 for sections of the LGE of *Rosa-rtTA;TRE-mCMV-H2B-GFP* embryos at E17.5 after exposure to 9TB-Dox at E9.5. Nuclei were stained with Hoechst 33342. Scale bar, 20 μm. Dashed lines indicate the ventricular surface. Arrowheads indicate GFP-retaining slowly dividing cells. **d** Quantification of Hey1 staining intensity for the indicated numbers of LRCs and non-LRCs (top 15% and bottom 60% of NPCs for H2B-GFP staining intensity, respectively) in the LGE at E17.5 for sections as in **c**. Data are expressed relative to the corresponding value for non-LRCs. Violin plots show the median (horizontal dashed line) and quartiles (horizontal dotted lines) (*n* = 1943 and 485 cells for non-LRCs and LRCs, respectively). Eight brain sections were analyzed per embryo, two-tailed Student’s *t* test. **e** RT and real-time PCR analysis of *Hey1* expression in cells positive for CD133 and GFP and negative for CD24 isolated by FACS from the neocortex of E17.5 embryos that had been subjected to in utero electroporation at E14.5 with plasmids for GFP alone (control) or together with p57 or p18. Data were normalized by the amount of β-actin mRNA, are expressed relative to the corresponding value for control, and are means ± SEM for *n* = 3 independent experiments for p57 or *n* = 5 independent experiments for p18, two-tailed paired *t* test.

Given the quantitative difference in Notch1 signaling between rapidly dividing and slowly dividing embryonic NPCs, we investigated whether there were any qualitative differences in signaling between these NPC populations by an examination of the expression levels of several Hes/Hey family members as typical Notch effectors. We performed an H2B-GFP retention analysis and isolated two fractions of CD133^+^CD24^–^ NPCs from the LGE at E16.5 by fluorescence-activated cell sorting (FACS) on the basis of H2B-GFP fluorescence intensity (top 8%, GFP^++^; middle 40% to 65%, GFP^+^) (Fig. 3a and Supplementary Fig. 4a). Reverse transcription (RT) and a real-time polymerase chain reaction (PCR) analysis revealed that the abundance of *Hey1* and *Hey2* mRNAs was significantly higher in slowly dividing (GFP^++^) NPCs than in rapidly dividing (GFP^+^) NPCs (Fig. 3b). The expression levels of *Hes1* and *Hes5* were also slightly higher in GFP^++^ NPCs than in GFP^+^ NPCs (Fig. 3b), but the fold differences in the expression of these genes between these two cell populations were not as great as were those in *Hey1* or *Hey2* expression (Supplementary Fig. 4b). Since Hey2 is much less abundant than Hey1 (Supplementary Fig. 2b, c), this suggests that Hey1 may distinguish slow cycling from rapid cycling NPCs. Indeed, IHC revealed that Hey1 protein levels were significantly higher in slowly dividing (top 15% H2B-GFP label-retaining) than rapidly dividing (bottom 60% H2B-GFP label retaining) NPCs (Ascl1- cells in the VZ) (Fig. 3c, d).

To determine whether the higher expression of Hey1 in slowly dividing NPCs is directly related to cell cycle inhibition, we inhibited the cell cycle of rapidly dividing NPCs in the neocortex by forced expression of p57 or p18. Overexpression of either p57 or p18 resulted in a significant increase in the level of *Hey1* mRNA (Fig. 3e), suggesting that cell cycle inhibition is sufficient to induce *Hey1* expression in NPCs.

### Hey1 plays a pivotal role in the maintenance of slowly dividing NPCs

**Fig. 4:**
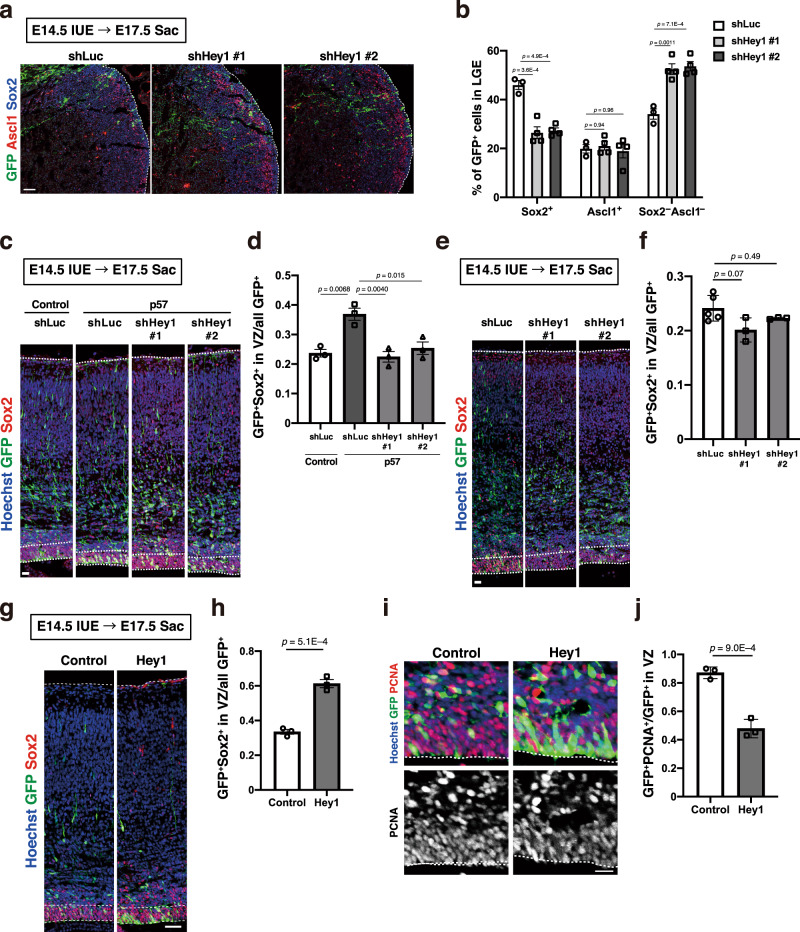
Hey1 contributes to the maintenance of slowly dividing NPCs. **a** Immunohistochemical analysis with antibodies to GFP, to Ascl1, and to Sox2 for the LGE at E17.5 after in utero electroporation at E14.5 with plasmids for GFP and either shLuc, shHey1 #1, or shHey1 #2. Scale bar, 100 μm. Dashed lines indicate the ventricular surface. **b** Quantification of the proportion of Sox2^+^, Ascl1^+^, or Sox2^−^Ascl1^−^ cells among all GFP^+^ cells in the LGE for sections as in **a**. Five brain sections were analyzed per embryo. Data are means ± SEM (*n* = 3, 4, and 4 embryos for shLuc, shHey1 #1, and shHey1 #2, respectively), one-way ANOVA followed by Scheffe’s multiple comparison test. **c**, **e** Immunohistochemical analysis with antibodies to GFP and to Sox2 for neocortical sections prepared at E17.5 from embryos that had been subjected to in utero electroporation at E14.5 with plasmids for control (shLuc) or Hey1 (#1 or #2) shRNAs and either GFP plus p57 (**c**) or GFP alone (**e**). Nuclei were stained with Hoechst 33342. Scale bars, 20 μm. Dashed lines the ventricular zone and the pial surface. **d**, **f** Quantification of the proportion of GFP^+^Sox2^+^ cells among all GFP^+^ cells for sections as in **c** and **e**, respectively. Five brain sections were analyzed per embryo. Data are means ± SEM for *n* = 3 embryos (**d**) or for *n* = 5, 3, and 3 embryos for shLuc, shHey1 #1, and shHey1 #2, respectively, one-way ANOVA followed by Scheffe’s multiple comparison test. **g**, **i** Immunohistochemical analysis with antibodies to GFP and to either Sox2 (**g**) or PCNA (**i**) for brain sections prepared at E17.5 after in utero electroporation with an expression plasmid encoding mouse Hey1 and GFP or with the corresponding plasmid encoding GFP alone (control) at E14.5. Nuclei were stained with Hoechst 33342. Scale bars, 50 μm (**g**) or 20 μm (**i**). Dashed lines indicate the ventricular zone and the pial surface. **h** Quantification of the proportion of GFP^+^Sox2^+^ cells among all GFP^+^ cells. Five brain sections were analyzed per embryo. Data are means ± SEM (*n* = 3 embryos), two-tailed Student’s *t* test. **j** Quantification of the proportion of GFP^+^PCNA^+^ cells among all GFP^+^ cells in the VZ. Five brain sections were analyzed per embryo. Data are means ± SEM (*n* = 3 embryos), two-tailed Student’s *t* test.

Hes1 and Hes5 play a pivotal role in maintenance of rapidly dividing neocortical NPCs during development^31,37,40^. Given the high expression level of Hey1 selectively among Hes/Hey family members in slowly dividing embryonic NPCs of the LGE, we next examined the possible role of Hey1 in the maintenance of NPCs in the dorsal LGE (dLGE). We confirmed that the introduction of two different short hairpin RNAs (shRNAs) by in utero electroporation at E14.5 resulted in a reduction of *Hey1* mRNA in NPCs isolated at E16.5 (Supplementary Fig. 5a). Such knockdown of Hey1 resulted in a reduction in the fraction of Sox2^+^ cells and an increase in that of Sox2^–^Ascl1^–^ (differentiated) cells among GFP^+^ (transfected) cells in the dLGE (Fig. 4a, b), suggesting that Hey1 facilitates the maintenance of slowly dividing NPCs in the dLGE.

We then asked whether Hey1 might play a role in the promotion of NPC maintenance mediated by cell cycle arrest. We found that knockdown of Hey1 prevented the increase in the fraction of Sox2^+^ (undifferentiated) cells among GFP^+^ (transfected) cells in the VZ at E17.5 induced by overexpression of p57 in the neocortex (Fig. 4c, d). Knockdown of Hey1 in the absence of p57 overexpression had no significant effect on the Sox2^+^ fraction (Fig. 4e, f), supporting the notion that Hey1 contributes to maintenance of the undifferentiated state of slowly dividing NPCs but not that of rapidly dividing NPCs. We then asked whether Hes1 and Hes5 might also play a role in the promotion of NPC maintenance mediated by cell cycle arrest. However, knockdown of Hes1 and Hes5 did not prevent the increase in the Sox2^+^ fraction induced by p57 overexpression (Supplementary Fig. 5b–e), suggesting that Hes1 and Hes5 are not required for the maintenance of slowly dividing NPCs.

We then examined the effect of Hey1 overexpression in rapidly dividing NPCs and found that expression of Hey1 in neocortical NPCs at E14.5 resulted in an increase in the Sox2^+^ fraction and a decrease in the PCNA^+^ fraction in the VZ at E17.5 (Fig. 4g–j). This suggests that high level expression of Hey1 is sufficient for promoting the maintenance of slowly dividing NPCs.

### Hey1 contributes to the emergence of slowly dividing NPCs in the LGE and of postnatal NSCs

**Fig. 5:**
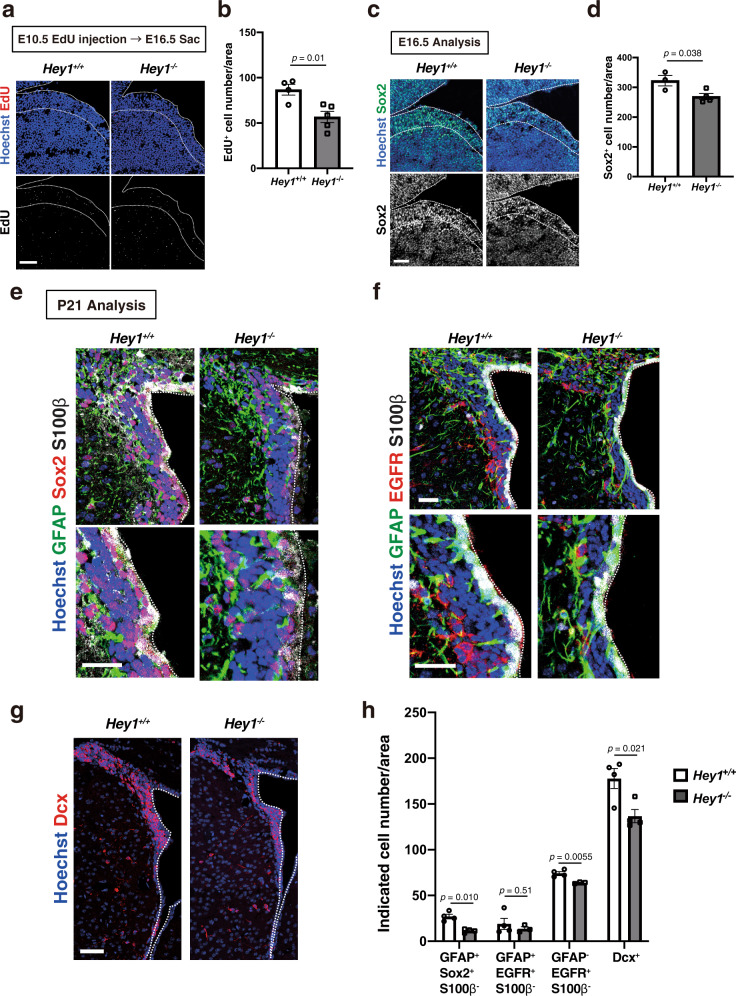
Hey1 knockout reduces the number of slowly dividing NPCs and postnatal NSCs. **a** EdU labeling for dLGE sections of Hey1 knockout and control embryos at E16.5 that had been exposed to EdU at E10.5. Nuclei were stained with Hoechst 33342. Scale bar 50 μm. Dashed lines indicate the border of the ventricular zone. **b** Quantification of the density of EdU^+^ cells in sections as in **a**. Five brain sections were analyzed per embryo. Data are means ± SEM (*n* = 4 or 5 for control and Hey1 knockout embryos, respectively), two-tailed Student’s *t* test. **c** Immunohistochemical analysis with antibodies to Sox2 for sections of the brain of control and Hey1 knockout mice at E16.5. Nuclei were stained with Hoechst 33342. Scale bars, 50 μm. Dashed lines indicate the border of the ventricular zone. **d** Quantification of the number of Sox2^+^ cells in sections as in **c**. Five brain sections were analyzed per embryo. Data are means ± SEM (*n* = 3 or 4 for control and Hey1 knockout embryos, respectively), two-tailed Student’s *t* test. **e**–**g** Immunohistochemical analysis with antibodies to GFAP, to Sox2, to S100β, to EGFR, and to Dcx (as indicated) for sections of the brain of control and Hey1 knockout mice at postnatal day (P) 21. Nuclei were stained with Hoechst 33342. Scale bars, 20 μm (**e**, **f**) or 50 μm (**g**). Dashed lines indicate the ventricular surface. **h** Quantification of the density of cells with the indicated marker phenotypes in the lateral wall of the SVZ for sections as in **e** through **g**. Five brain sections were analyzed per sample. Data are means ± SEM for *n* = 4 and 4 (**e**, **g**) or *n* = 4 and 3 (**f**) control and Hey1 knockout mice, respectively, two-tailed Student’s *t* test.

Given that most postnatal and adult NSCs in the lateral wall of the SVZ are derived from slowly dividing embryonic NPCs^15,16^, we asked whether Hey1 regulates the emergence of this embryonic population in the dLGE as well as postnatal NSCs in the corresponding area of the SVZ. Using Hey1 knockout mice, we examined whether Hey1 affects the abundance of slowly dividing NPCs in the dLGE. We detected these cells on the basis of 5-ethynyl-2′-deoxyuridine (EdU) retention at E16.5 after its injection at E10.5. We indeed found that the number of EdU-retaining (slowly dividing) cells and Sox2^+^ (undifferentiated) cells in the dLGE (dorsal one-fourth of the LGE at the level of the rostral one-third of the rostral-caudal axis) were significantly lower for Hey1 knockout mice than for control mice (Fig. 5a–d), implicating Hey1 in either establishment or maintenance of this slowly dividing embryonic NPC population. We next examined the effect of *Hey1* deletion on postnatal NSCs in the corresponding SVZ area (dorsal one-third of the lateral wall at the level of the rostral-caudal axis, +0.9 to +0.1 mm relative to the bregma) where around 60% of NSCs defined as GFAP^+^Vcam1^+^ cells expressed Hey1 at high levels at P15 (Supplementary Fig. 6b, c). The number of NSCs—as defined by the presence of the NSC markers GFAP and Sox2 as well as the absence of the ependymal marker S100β—was significantly reduced in the SVZ of Hey1 knockout mice compared with that of control mice (Fig. 5e, h). The numbers of NSC progeny, including transit amplifying cells (GFAP^–^EGFR^+^S100β^–^) and neuroblasts (Dcx^+^), were also significantly reduced in this region by deletion of *Hey1* (Fig. 5f–h). Together, these results indicated that Hey1 plays a key role in the effective establishment or maintenance of the postnatal NSC population in this area of the SVZ.

### *Hey1* mRNA is more stable than *Hes1* and *Hes5* mRNAs in NPCs

**Fig. 6:**
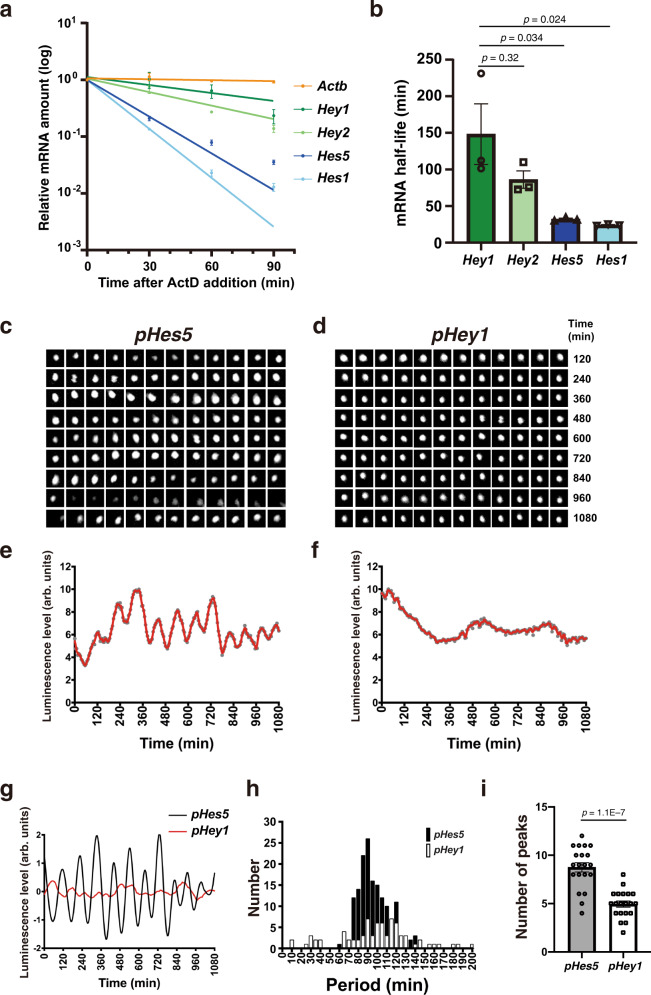
The *Hey1* promoter confers nonoscillatory expression dynamics. **a** RT and real-time PCR analysis of *Hes/Hey* and *Actb* (β-actin) mRNA levels in cultured NPCs incubated in the presence of actinomycin D (5 µM) for the indicated times. The amount of each mRNA was expressed relative to that at time 0 (no actinomycin D treatment). Data are means ± SEM (*n* = 3 independent experiments). **b** Half-lives of *Hes/Hey* mRNAs determined from the data in **a**, one-way ANOVA followed by Scheffe’s multiple comparison test. **c**, **d** The pHes5-NLS-Ub-Luc2-Hes5-3′UTR or pHey1-NLS-Ub-Luc2-Hey1-3′UTR plasmids were introduced into cultured NPCs by electroporation together with expression vectors for NICD1 and mCherry. Representative bioluminescence images of individual mCherry^+^ cells are shown. **e**, **f** Quantification of bioluminescence for cells as in **c** and **d**, respectively. Data are expressed in arbitrary units (arb. units). **g** Detrended and smoothed data for **e** and **f**, respectively. **h** Distribution of oscillation period for *Hes5* or *Hey1* promoter activity determined from bioluminescence measurements (*n* = 20 cells for each sample). **i** Number of detected peaks for bioluminescence driven by the *Hes5* or *Hey1* promoters. Data are means ± SEM (*n* = 20 cells for each sample), two-tailed Student’s *t* test.

Given that Hey and Hes proteins may have different roles in the maintenance of slowly dividing and rapidly dividing NPCs, respectively, we searched for differences in the properties of Hey and Hes family members that might account for such differential roles. *Hey1* mRNA was previously found to be more stable than *Hes5* mRNA in the chick inner ear^53^, so we investigated whether such difference might exist in mouse NPCs. Exposure of NPCs isolated from the mouse GE to the transcriptional inhibitor actinomycin D revealed that the half-lives of *Hey1* and *Hey2* mRNAs were 148 ± 42 and 86 ± 12 min, respectively (Fig. 6a, b), with these times being substantially longer than those for *Hes1* and *Hes5* mRNAs at 24 ± 0.6 and 32 ± 1.4 min, respectively (Fig. 6a, b), consistent with previously determined values of ~25 min^41,54^. Our results thus indicated that *Hey1* and *Hey2* mRNAs are more stable than *Hes1* and *Hes5* mRNAs in mouse NPCs.

### The *Hey1* promoter confers non-oscillatory expression dynamics

Transcript stability is predicted to be a key determinant of the expression dynamics of Hes1 and Hes5. Both mathematical models and experimental results^55–57^ suggest that an oscillatory expression pattern requires a short half-life of each mRNA (<50 min). In contrast to the *Hes* genes, the expression dynamics of *Hey1* have remained elusive. The *Hey1* promoter contains an N-box—a sequence that mediates transcriptional suppression by Hes/Hey proteins—suggesting it may suppress its own expression. Nevertheless, the stability of *Hey1* mRNA may hamper the establishment of oscillatory dynamics. We used cultured NPCs to measure activity of the mouse *Hey1* promoter (–2.8 to 0 kb relative to the transcription start site (TSS)), which contains five RBP-J binding sites (TGGGAA or TTCCCAC) and four N-boxes (CACCAG or CTGGTG), and is activated by NICD1–4^58^ and repressed by Hey1^59^. For comparison, we monitored the activity of the mouse *Hes5* promoter (–3.0 to 0 kb relative to the TSS), which contains seven RBP-J sites and three N-boxes. Reporter constructs in which the *Hey1* or *Hes5* promoter drives expression of a short-lived (ubiquitinated) form of firefly luciferase (Ub-Luc) were introduced into NPCs, and luciferase levels were visualized by time-lapse bioluminescence imaging over a prolonged period. In the presence of co-expressed NICD1, which increased the activity of both promoter constructs, the activity of the *Hes5* promoter oscillated with a period of ~90 min (Fig. 6c, e, g, h; Supplementary movie 1), consistent with previous observations^42^. In contrast, *Hey1* promoter activity did not show an obvious oscillatory pattern (Fig. 6d, f, g, h; Supplementary movie 2). Indeed, the number of detected peaks for expression of the *Hey1* reporter was significantly lower than that for expression of the *Hes5* reporter (Fig. 6i), suggestive of a stable or constant pattern of *Hey1* expression. Moreover, the time interval between consecutive peaks (period) for expression of the *Hey1* reporter varied widely (Fig. 6h). Indeed, the variation of period values for the *Hey1* reporter was significantly larger than that for the *Hes5* reporter (*F* test, *p* < 0.001), again indicative of a non-oscillatory pattern of *Hey1* expression. These results suggested that the expression dynamics of the *Hey1* promoter are non-oscillatory and are more constant than are those of the *Hes5* promoter. Combined with the slower degradation of Hey1 transcript, this would lead to a constant level of Hey1 RNA and presumably Hey1 protein.

### Suppression of proneural gene expression in slowly dividing embryonic NPCs

**Fig. 7:**
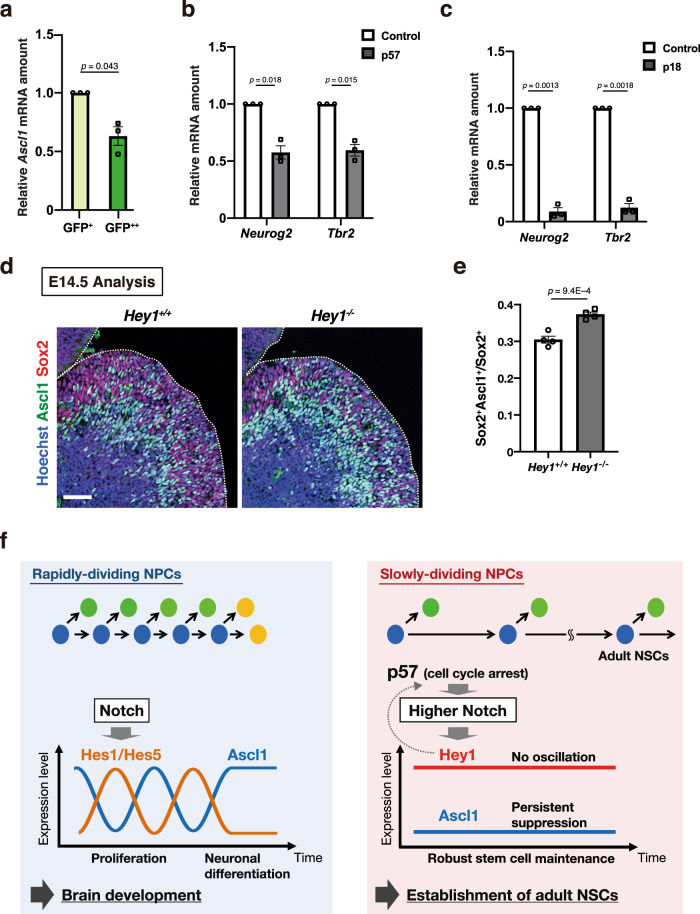
Suppression of proneural gene expression in slowly dividing NPCs. **a** RT and real-time PCR analysis of *Ascl1* mRNA in NPCs isolated from the LGE of *Rosa-rtTA;TRE-mCMV-H2B-GFP* embryos at E16.5 and sorted on the basis of H2B-GFP retention after exposure to 9TB-Dox at E9.5. Data were normalized by the amount of β-actin mRNA, are expressed relative to the corresponding value for GFP^+^ cells, and are means ± SEM (*n* = 3 independent experiments), two-tailed paired *t* test. **b**, **c** RT and real-time PCR analysis of *Neurog2* and *Tbr2* mRNAs in cells positive for CD133 and GFP and negative for CD24 isolated by FACS from the neocortex of E17.5 embryos that had been subjected to in utero electroporation at E14.5 with plasmids for GFP alone (control) or together with p57 (**b**) or p18 (**c**). Data were normalized by the amount of β-actin mRNA, are expressed relative to the corresponding value for control, and are means ± SEM (*n* = 3 independent experiments), two-tailed paired *t* test. **d** Immunohistochemical analysis with antibodies to Ascl1 and to Sox2 for sections of the brain of control and Hey1 knockout mice at E14.5. Nuclei were stained with Hoechst 33342. Scale bar, 50 μm. Dashed lines indicate the ventricular surface. **e** Quantification of the proportion of Ascl1^+^ cells among Sox2^+^ cells in the dLGE for sections as in **d**. Data are means ± SEM (*n* = 4 embryos), two-tailed Student’s *t* test. **f** Model for the division of labor of Notch downstream effectors that can select the fate of “rapidly dividing” (oscillating Hes1/Hes5) versus “slowly dividing” (tonic Hey1) pools of embryonic NPCs, respectively.

The high levels of expression of NICD1 and Hey1, and the evidently constant pattern of *Hey1* expression, may contribute to the robust suppression of proneural genes in NPCs. We compared the levels of proneural gene expression between slowly dividing and rapidly dividing NPCs isolated from the LGE of mouse embryos at E16.5. This analysis revealed that the amount of *Ascl1* mRNA was significantly lower in H2B-GFP–retaining (slowly dividing) NPCs than in non-retaining (rapidly dividing) NPCs (Fig. 7a). Furthermore, we found that the overexpression of either p57 or p18 significantly reduced the levels of *Neurog2* and *Tbr2* mRNAs in neocortical NPCs at E17.5, with the latter mRNA representing a marker of intermediate neuronal progenitors in the neocortex (Fig. 7b, c). These results thus suggested that cell cycle inhibition reduces the levels of proneural gene expression in NPCs and that this regulation contributes to the robust maintenance of slowly dividing NPCs.

We then asked whether Hey1 might contribute to the suppression of Ascl1 expression in the dLGE. We indeed found that the proportion of Ascl1^+^ cells among Sox2^+^ (undifferentiated) cells in the dLGE was significantly higher in Hey1 knockout mice than in control mice (Fig. 7d, e). This result points to a function of Hey1 in the suppression of Ascl1 in slowly dividing NPCs.

## Discussion

Cell cycle inhibition has been implicated in the ontogeny of NSCs in the SVZ of adult mice, but the underlying mechanisms have been unclear. In this study, we show that the slowly dividing subpopulation of embryonic NPCs, which include the precursors of adult NSCs, shows a marked upregulation and higher levels of active Notch1 and Hey1 compared with the rapidly dividing embryonic NPCs that contribute to brain development. In support, forced cell cycle inhibition activated the Notch-Hey1 signaling pathway in NPCs. Hey1 has a selective role in promoting the undifferentiated state of slowly dividing NPCs, and its deletion impaired the emergence of slowly dividing embryonic NPCs (E16.5) and early postnatal NSCs (P21) in the lateral wall of the SVZ, without a noticeable effect on gross brain development (Supplementary Fig. 6a). Remarkably, the *Hey1* promoter has a non-oscillatory expression pattern in contrast to the oscillatory *Hes5* promoter. Taken together, we propose a novel division-of-labor model for embryonic neurogenesis (Fig. 7f). In one arm, high levels of Notch signaling and stable non-oscillatory *Hey1* expression in slowly dividing embryonic NPCs facilitate their stable maintenance to postnatal and adult stages through the persistent suppression of proneural gene expression. In another arm, lower levels of Notch and Hes1 and Hes5 are associated with rapidly dividing NPCs for oscillatory expression and recruitment into neurogenic programs for building the basic brain plan during development. Together, these two Notch effector arms regulate development of the tight balance between developmental and adult pools of unique progenitors while specifying their unique properties.

How does cell cycle arrest induce activation of Notch1 signaling? The level of NICD may provide an important clue. We found that the level of NICD1, but not that of *Notch1* mRNA, was significantly higher in slowly dividing NPCs than in rapidly dividing NPCs (Fig. 2f, g and Supplementary Fig. 8a). Thus, mRNA translation or activating processing or degradation of Notch1 protein might differ between these two cell populations. Cdk1 and Cdk2 phosphorylate NICD1 and promote its Fbw7-dependent degradation by the ubiquitin-proteasome system in chick somites and human cell lines^60^. With *FucciG1* reporter mice, we found that most slowly dividing NPCs are in late G_1_ phase (Supplementary Fig. 7), when Cdk1 and Cdk2 (as well as Cdk4) are supposed to be inactive, suggesting that cell cycle inhibition may increase the amount of NICD1 in slowly dividing NPCs by suppressing Cdk-dependent degradation. We also found that overexpression of p57 increased the expression of *Lunatic fringe* (*Lfng*) and *Eya1* (Supplementary Fig. 8c). Given the role of Lfng in glycosylation and activation of Notch^61^ and that of Eya1 in dephosphorylation and prevention of NICD1 degradation^62^, Lfng and Eya1 may participate in activation of Notch signaling in slowly dividing NPCs. It is also possible that cell cycle inhibition prevents the dilution of NICD1 associated with cell division. However, we found that forced cell cycle inhibition at late G_1_ phase by overexpression of p57 or p18 in cultured NPCs did not increase Notch signaling activity (data not shown), in contrast to their effect in vivo. Evidently, other mechanisms in vivo appear to control the upregulation of NICD1 in slowly dividing NPCs in addition to the suppression of Cdk-mediated degradation and/or NICD1 dilution.

How is Hey1 more effectively upregulated than Hes1/5 in slowly dividing NPCs? In principle, different levels of NICD1 per se might have differential effects on target gene induction^63^. However, the temporal (pulsatile or sustained) dynamics of Notch activity, rather than the absolute levels, may determine the pattern of target gene induction in cell culture dependent on different Notch ligands. For example, pulsatile Notch1 activation triggered by Dll1 can result in *Hes1* induction, whereas sustained Notch1 activation elicited by Dll4 may favor *Hey1* induction^64^. If cell cycle inhibition triggers Notch1 activation in slowly dividing NPCs, as we believe, we expect this effect to be sustained, which may explain the preferential induction of *Hey1* rather than *Hes1*. It is also possible that different Notch isoforms differentially signal in slowly dividing versus rapidly dividing NPCs. For instance, Notch2 and Notch3 have been shown to play a key role in the maintenance of quiescent adult NSCs, whereas Notch1 appears to contribute to the activation of these cells^17,21,65,66^. Indeed, we found that *Notch2* and *Notch3* but not *Notch1* are expressed at a higher level in slowly dividing NPCs than in rapidly dividing NPCs of the mouse embryonic LGE (Supplementary Fig. 8a), and are significantly upregulated by overexpression of p57 (Supplementary Fig. 8b). Thus, Notch2 and Notch3 may be involved in *Hey1* induction, although the levels of induction of *Hey1, Hes1*, and *Hes5* mRNA were not overtly different between NICD1, NICD2, and NICD3 when overexpressed in cultured NPCs (Supplementary Fig. 8d). Further studies will be required to clarify which Notch receptors, Notch ligands and Notch protein modifications, as well as whether and how the epigenetic state of the *Hey1* gene, contribute to the preferential induction of *Hey1* and establishment of the slowly dividing embryonic NPC population.

The effect of stationary (e.g., tonic) expression dynamics of Notch effectors on the suppression of the neurogenic program is an important implication of our findings. Oscillatory dynamics of Hes1 and Hes5 expression have been shown to induce a low level and oscillatory dynamics of the expression of neurogenic target genes such as *Ascl1* and *Neurog2* in NPCs^42,43^. The expression pattern of *Ascl1* appears to be essential for promotion of cell proliferation and inhibition of premature differentiation. Indeed, ablation of Ascl1 results in cell cycle arrest in addition to suppression of neuronal differentiation, whereas expression of Ascl1 at a high level promotes premature neuronal (and oligodendrocytic) differentiation, in embryonic NPCs and adult NSCs^28,42,67–69^. Thus, the constant expression of Hes/Hey family members at a high level may result in the persistent suppression of *Ascl1* expression, cell cycle arrest, and robust maintenance of the undifferentiated state of NPCs/NSCs. Of interest, a recent study in the adult mouse SVZ and hippocampal dentate gyrus found that Hes1 exhibits a high level and oscillatory pattern of expression in quiescent NSCs and, together with other Hes/Hey family members (Hes3, Hes5, and Hey1), induces the persistent suppression of *Ascl1* in and promotes maintenance of these cells^70^. However, in the embryonic brain, Hey1 knockdown, but not Hes1 knockdown, partially compromised the undifferentiated state in slowly dividing NPCs (Fig. 4 and Supplementary Fig. 5). Indeed, in the Hey1 knockout there was a reduced number of early postnatal NSCs at P21 (Fig. 5e–h). These results suggest that Hey1, but not Hes1, plays a pivotal role in the establishment of slowly dividing embryonic NPCs and early postnatal NSCs, whereas, at later stages, additional mechanisms result in a high level of Hes1 expression, which further promotes the maintenance of adult NSCs.

In summary, we found that cell cycle inhibition activates Notch signaling and increases Hey1 expression in slowly dividing embryonic NPCs for the maintenance of the undifferentiated state and ontogeny of adult NSCs and postnatal neurogenesis. There are at least three broader implications of these findings beyond basic mechanisms of neurogenesis. First, Hey1-deficient mice are reported to have psychiatry-related phenotypes such as anxiety-like behavior^71,72^, and it will be of interest to investigate the contribution of the current mechanisms to such phenotypes. Second, the suppression of proneural genes may not be the only outcome of Notch-Hey1 signaling. Notch activation can induce the expression of carnitine palmitoyltransferase 1a (Cpt1a) and fatty acid oxidation in quiescent endothelial cells^73^, and Cpt1a mediates the maintenance of adult NSCs^74^. Therefore, Cpt1a-dependent metabolic changes may be a further consequence of cell cycle inhibition and activation of Notch signaling in slowly dividing NPCs. Finally, Hey1 has also been implicated in satellite cell maintenance in adult muscle^75^, suggesting the possibility that the link between cell cycle arrest and Notch-Hey1 signaling may play a role in the long-term maintenance of various adult tissue stem cells.

## Methods

### Mice

*FucciG1* mice^76^ (RBRC02707) and Hey1 knockout mice^77^ (RBRC01151) were obtained from the RIKEN Bioresource Center [http://www.brc.riken.jp/lab/animal/en]. *Rosa26-rtTA* (#006965) and *TRE-mCMV-H2B-GFP* mice (#005104)^78^ were obtained from The Jackson Laboratory. *pHes1-d2EGFP* mice were previously described^79^. ICR mouse embryos (Japan SLC) were subjected to in utero electroporation for analysis. All mice were maintained in a temperature- and relative humidity–controlled (23° ± 3 °C and 50 ± 15%, respectively) environment with a normal 12-h-light, 12-h-dark cycle. They were housed two to six per sterile cage (Innocage, Innovive; or Micro BARRIER Systems, Edstrom Japan) with chips (PALSOFT, Oriental Yeast; or PaperClean, SLC Japan), and with irradiated food (CE-2, CLEA Japan) and filtered water available ad libitum. Mouse embryos were isolated at various ages, with E0.5 being considered the time of vaginal plug appearance. All animals were maintained and studied according to protocols approved by the Animal Care and Use Committee of The University of Tokyo.

### Plasmid constructs and RNA interference

For the construction of shRNA vectors, oligonucleotides corresponding to the target coding sequence and its complementary sequence were inserted into the pSIREN vector. The targeting sequences were as follows:

shHey1 #1, 5′-GCAGGGATCTGCTAAGCTAGA-3′;

shHey1 #2, 5′-GCAGCAAGCAAGACAGTTATG-3′;

shHes1, 5′-GGCATTCCAAGCTAGAGAAGG-3′;

shHes5, 5′-GCTGTTCTGAAGGCTTCTAAT-3′;

shLuc (control), 5′-GTGCGTTGCTAGACCAAC-3′.

For the Hey1 reporter construct, 2.8-kb promoter and 1.0-kb of downstream sequence of *Hey1* were used. For the Hes5 reporter construct, ﻿3-kb of the 5′-flanking sequence and 2-kb of downstream sequence were used as previously described^42^. The plasmids pCDNA3-p57 and pCDNA3-p57ΔCKI mutant were used as previously described^16^. The plasmids pCAGGS-p18 and pEFBOS-NICD1 were kindly provided by F. Matsuzaki and M. Nakafuku, respectively. EGFP sequence was inserted into pCS4 and pCAGEN to generate pCS4-EGFP and pCAGEN-EGFP, respectively. mCherry sequence was inserted into pCAGEN to generate pCAGEN-mCherry. The plasmids pCAG2IG-NICD1 and pCAG2IG-NICD3 were used as previously described^21^. 3XFlagNICD2 was purchased from Addgene (plasmids #20184) and inserted into pCAG2IG to generate pCAG2IG- NICD2.

### Immunofluorescence analysis

For immunohistochemical staining of coronal brain sections, mice were anesthetized by Pentobarbital (Nacalai tesque) and then transcardially perfused with ice-cold 4% paraformaldehyde (Merck) in phosphate-buffered saline (PBS). The brain was then removed, exposed to the same fixative at 4 °C for 1 to 3 h, equilibrated with 30% (w/v) sucrose in PBS, embedded in OCT compound (Tissue TEK), and frozen. Coronal cryosections (thickness of 12–16 μm) were exposed to Tris-buffered saline containing 0.1% Triton X-100 and 3% bovine serum albumin (blocking buffer) for 2 h at room temperature, incubated first overnight at 4 °C with primary antibodies in blocking buffer and then for 2 h at room temperature with Alexa Fluor–conjugated secondary antibodies (Thermo Fisher Scientific) in blocking buffer, and mounted in Mowiol (Calbiochem). Fluorescence images were obtained with a laser confocal microscope (Leica TCS-SP5 or Zeiss LSM 880) and were processed with the use of LAS AF (Leica), ZEN (Zeiss), Photoshop CS (Adobe), and Image J (U.S. National Institutes of Health) software.

### Antibodies

Antibodies for immunostaining included chicken anti-GFP (1:1000 dilution, Abcam, Cat# ab13970), rat anti-GFP (1:1000, Nacalai Tesque, Cat# GF090R), anti-Sox2 (1:200, Cell Signaling Technology, Cat# 3728), anti-RFP (1:1000, MBL, Cat# PM005), anti-Fabp7 (BLBP, 1:500, Millipore, Cat# ABN14), mouse anti-S100β (1:200, Sigma-Aldrich, Cat# S2657), rabbit anti-S100β (1:500, Abcam, Cat# ab52642), anti-NICD1 (1:200, Cell Signaling Technology, Cat# 4147), anti-GFAP (1:1000, Abcam, Cat# ab4674), anti-Hey1 (1:200, Millipore, Cat# AB5714), anti-Ascl1 (1:200, BD Biosciences, Cat# 556604), anti-PCNA (1:500, Millipore, Cat# NA03), anti-Dcx (1:1000, Abcam, Cat# ab18723), anti-EGFR (1:500, Fitzgerald, Cat# 20-ES04), anti-Vcam1 (1:500, BD Biosciences, Cat# 550547), Hoechst 33342 (1:10000, Molecular Probes).

### Quantification of immunofluorescence analysis

Regarding quantification of immunofluorescent images, we analyzed more than four brain sections per sample, and more than three samples per experiment. The images were acquired on the same day within a short time for reducing the day-to-day variation of microscopic detection, and within the range below saturation of image acquisition. For quantification of the results in Fig. 2a, c, the region of interest (ROI) was manually created based on the RFP signal, and GFP intensity was quantified in each ROI. For quantification of the results in Figs. 2f and 3c, ROI was created using Hoechst signal by Image J software, and the immunofluorescent intensity of NICD1 and Hey1 was quantified in each ROI. The signal intensity was normalized by the mean value of corresponding signal intensities within non-LRCs. We determined the positivity of GFAP regarding the judgement of NSCs in Fig. 5 by the presence of a GFAP-harboring fiber that surrounds the nucleus. We also added information on the numbers of samples and sections in each experiment in the corresponding figure legend.

### H2B-GFP retention analysis

The expression of an H2B-GFP fusion protein was transiently induced at E9.5 by the injection of 9-*tert*-butyldoxycycline (9TB-Dox, 0.25 mg) into pregnant *Rosa-rtTA;TRE-mCMV-H2B-GFP* mice, and slowly dividing NPCs were detected as H2B-GFP–retaining NPCs as previously described^16^.

### Administration of EdU

For the identification of slowly dividing embryonic NPCs, pregnant mice were injected intraperitoneally with EdU (5 mg per kg body weight) four times at 3-h intervals at E10.5. EdU was detected with the use of a Click-iT EdU Imaging Kit (Invitrogen).

### In utero electroporation

Plasmid DNA was introduced into NPCs of the developing mouse embryonic neocortex and GE as previously described^80,81^. In brief, plasmid DNA was injected into the lateral ventricle at the indicated developmental stages, electrodes were positioned at the flanking ventricular regions, and four to eight 50-ms pulses of 33 to 45 V were applied at intervals of 950 ms with the use of an electroporator (CUY21E, Tokiwa Science). The uterine horn was returned to the abdominal cavity to allow continued development of the embryos. The pCS4-EGFP, pCAGEN-EGFP, and pCAGEN-mCherry plasmids were used to identify successfully electroporated cells.

### FACS

The LGE of *Rosa-rtTA;TRE-mCMV-H2B-GFP* mice at E16.5 or the neocortex of electroporated embryos at E17.5 was dissected and subjected to enzymatic digestion with a papain-based solution (Sumitomo Bakelite), and the dissociated single cells were isolated and incubated for 20 min at room temperature with PBS containing phycoerythrin- and Cy7-conjugated antibodies to CD133 (1:200 dilution, BioLegend, 141210) and allophycocyanin-conjugated antibodies to CD24 (1:200 dilution, BioLegend, 101814). Cells were directly subjected to fluorescence-activated cell sorting (FACS) with a FACS Aria instrument (Becton Dickinson). Debris and aggregated cells were removed by gating on the basis of forward and side scatter. NPCs are collected on the basis of the presence of NPC marker CD133 (also known as prominin)^82^ and the absence of the neuronal marker CD24^83^. Gates were set as described previously^84^. Data were analyzed with the use of FlowJo software.

### RT and real-time PCR analysis

Total RNA was isolated from sorted NPCs with the use of RNAiso plus (Takara), and up to 0.5 μg of the RNA was subjected to RT with the use of ReverTra Ace qPCR Master Mix (Toyobo). The resulting cDNA was subjected to real-time PCR analysis in a LightCycler 480 instrument (Roche) with any of KAPA SYBR FAST for LightCycler 480 (Kapa Biosystems), Thunderbird SYBR qPCR mix (Takara), and QuantiNova SYBR Green PCR kit (Qiagen). The amount of target mRNA was normalized by that of β-actin mRNA. The sequences of PCR primers are provided in Supplementary Table 1.

### Primary NPC culture

Primary NPC culturing was performed as described previously^85^. Primary NPCs were obtained from the GE of ICR mouse embryos at E11.5. The dissected GE was subjected to enzymatic digestion with a papain-based solution (Sumitomo Bakelite), and the dissociated single cells were cultured for 3 days in Dulbecco’s modified Eagle’s medium–F12 (1:1) supplemented with B27 (Invitrogen), fibroblast growth factor 2 (20 ng/ml, Invitrogen), and epidermal growth factor (20 ng/ml, Invitrogen) to allow the formation of neurospheres in suspension. The neurospheres were then dissociated into single cells and plated on poly-D-lysine–coated dishes.

### RNA degradation assay

Cultured NPCs were exposed to 5 µM actinomycin D (Sigma) for 0, 30, 60, or 90 min, after which total RNA was isolated from the cells and the amounts of target mRNAs determined. The mRNA level at each time point was normalized by that at time 0 (no actinomycin D treatment). Curve fitting was performed with Prism software. The degradation rate (*k*) of each mRNA was determined according to the equation *y* = e^–*kx*^, and mRNA half-life (*t*_1/2_) was calculated with the equation *t*_1/2_ = ln(2)/*k*.

### Bioluminescence imaging of cultured NPCs

Dissociation culture of neural progenitors and bioluminescence imaging were performed as described^42^. In brief, plasmid DNA was introduced into cultured NPCs by nucleofection with the use of an AAD-1001 device (Amaxa). The cells ﻿were plated on 35-mm glass-bottom dishes ﻿and incubated at 37°C under 5% CO_2_. 1 mM luciferin (Nacalai Tesque, 0149385) was then added to the culture medium. Bioluminescence images were acquired with an ﻿upright microscope (IX83, Olympus) and a cooled CCD camera (iKon-M DU934P-BV, Andor). The filters and camera control were adjusted automatically with software (MetaMorph, Universal Imaging). Image analysis was performed using ImageJ software and custom plug-ins, as described previously^42,86^. Custom plugins is available at [https://github.com/aisomur/genes_dev_2017].

### Neuro2a cell culture and transfection

Neuro2a cells were maintained in Dulbecco’s modified Eagle’s medium (DMEM)—high glucose (Sigma-Aldrich) supplemented with 10% FBS (Gibco) and 1% penicillin-streptomycin (Invitrogen). They were transfected with the use of Lipofectamine 2000 following manufacturer’s instructions (Thermo Fisher Scientific).

### RNA sequencing analysis

Cells positive for CD133 and GFP and negative for CD24 were isolated by FACS from the neocortex of E17.5 embryos that had been subjected to in utero electroporation at E14.5 with plasmids for GFP alone (control) or together with p57 as shown in Supplementary Fig. 2. Purified RNA was used for the library construction for the RNA-Seq analysis. Template preparation was conducted using Illumina TruSeq stranded mRNA library kit. Constructed template was used for the deep sequencing analysis on the Illumina platform by 100-base paired-end read. Approximately, 40 million sequences were obtained for the RNA-seq analysis. Sequences reads were mapped to the reference mouse genome (mm10) with the use of Hisat2^87^. Only uniquely mapped and “deduplicated” reads with no base mismatch were used. *Cdkn1c* (p57) gene and any genes that were not expressed in at least three samples with reads >10 were excluded from further analysis. Reads were normalized by TMM (weighted trimmed mean of M-values) normalization^88^ as implemented in the R package ‘edgeR’^89^. Differential gene expression analysis between representative samples was performed using the R package ‘edgeR’^89^. Processed data of RNA sequence was shown in Supplementary Data 1. Gene clustering analysis based on expression patterns was performed with the SciPy Python package. Ontology and pathway enrichment analysis was conducted with DAVID software^90^.

### GSEA analysis

Normalized gene expression from RNA-seq was used for GSEA analysis. Enrichment of signature genes was assessed using a GSEA software^91^. Each gene set used for GSEA analysis was shown in Supplementary Data 2.

### Statistical analysis

Data are presented as mean ± SEM or as median and interquartile range, as indicated, and they were compared with the two-tailed Student’s *t* test, the two-tailed paired *t* test, the Mann–Whitney test, one-way analysis of variance (ANOVA) followed by Scheffe’s multiple comparison method, or by an *F* test. A *p* value of <0.05 was considered statistically significant.

### Reporting summary

Further information on research design is available in the Nature Research Reporting Summary linked to this article.

## Supplementary information

Supplementary Information

Description of Additional Supplementary Files

Supplementary Data 1

Supplementary Data 2

Supplementary Movie 1

Supplementary Movie 2

Reporting Summary

## Data Availability

The sequence data have been deposited in the DNA Data Bank of Japan (DDBJ) Sequence Read Archive under the following IDs: DRA010678. Source data are provided with this paper. All relevant data are available on request.

## Source data

Source Data
