# Supplementary material for: Cell cycle arrest determines adult neural stem cell ontogeny by an embryonic Notch-nonoscillatory Hey1 module
Source: Nat Commun. 2021 Nov 12;12:6562. doi: 10.1038/s41467-021-26605-0 (PMC8589987; doi:10.1038/s41467-021-26605-0)
Supplement: Supplementary file 1 — Supplementary Information [file 41467_2021_26605_MOESM1_ESM.pdf]

Supplementary Information

Cell cycle arrest determines adult neural stem cell ontogeny by an embryonic Notch-nonoscillatory *Hey1* module

Harada et al.

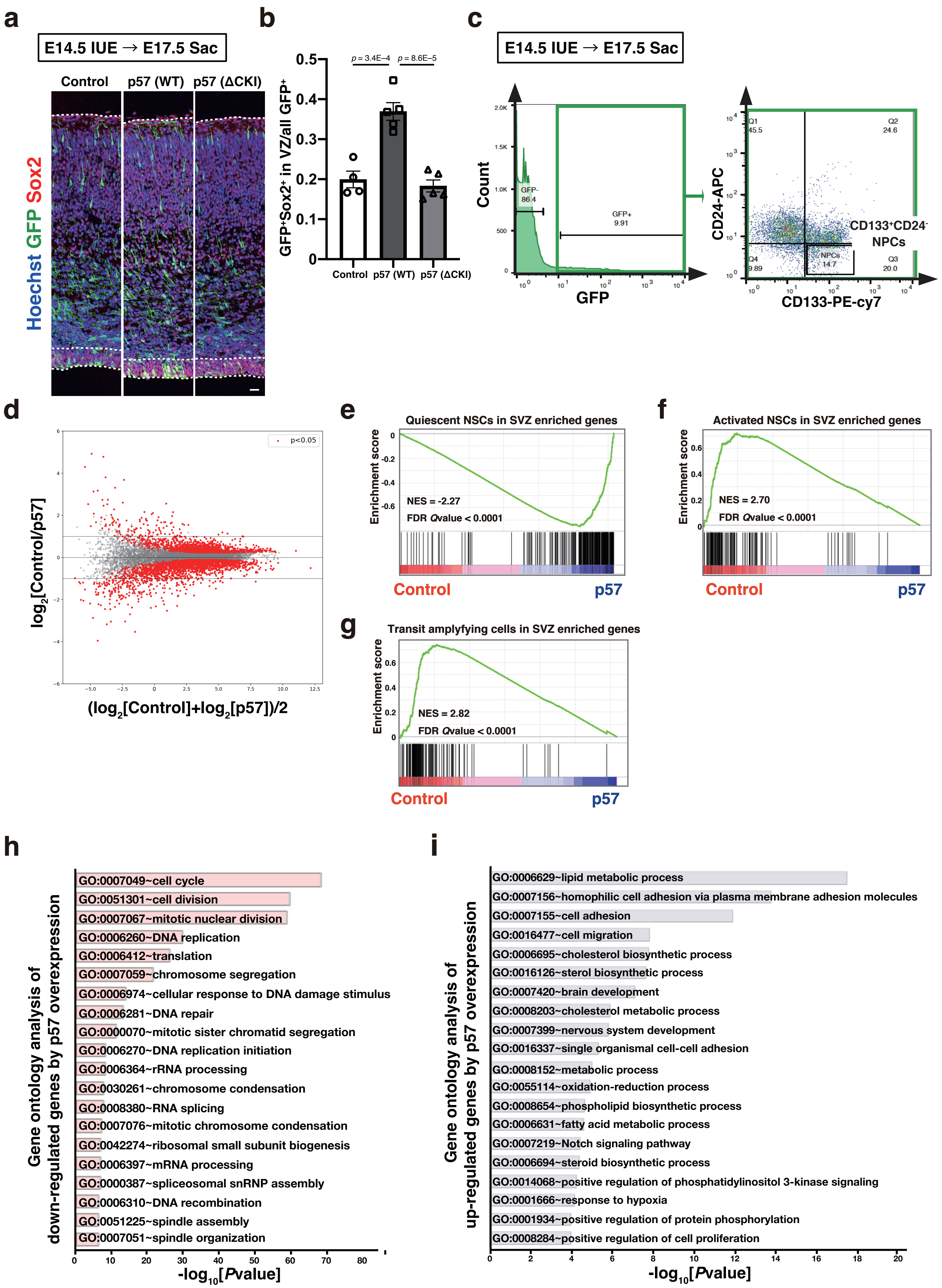
a
E14.5 IUE → E17.5 Sac
Control
p57 (WT)
p57 (ΔCKI)
Hoechst GFP Sox2
b
p = 3.4E–4
p = 8.6E–5
GFP+Sox2+ in VZ/all GFP+
Control
p57 (WT)
p57 (ΔCKI)
c
E14.5 IUE → E17.5 Sac
Count
GFP-
86.4
GFP+
9.91
GFP
CD24-APC
Q1
45.5
Q2
24.6
Q4
9.89
Q3
20.0
NPCs
14.7
CD133+CD24-
NPCs
CD133-PE-cy7
d
p<0.05
log2[Control/p57]
(log2[Control]+log2[p57])/2
e
Quiescent NSCs in SVZ enriched genes
Enrichment score
NES = -2.27
FDR Qvalue < 0.0001
Control
p57
f
Activated NSCs in SVZ enriched genes
Enrichment score
NES = 2.70
FDR Qvalue < 0.0001
Control
p57
g
Transit amplyfying cells in SVZ enriched genes
Enrichment score
NES = 2.82
FDR Qvalue < 0.0001
Control
p57
h
Gene ontology analysis of down-regulated genes by p57 overexpression
GO:0007049~cell cycle
GO:0051301~cell division
GO:0007067~mitotic nuclear division
GO:0006260~DNA replication
GO:0006412~translation
GO:0007059~chromosome segregation
GO:0006974~cellular response to DNA damage stimulus
GO:0006281~DNA repair
GO:0000070~mitotic sister chromatid segregation
GO:0006270~DNA replication initiation
GO:0006364~rRNA processing
GO:0030261~chromosome condensation
GO:0008380~RNA splicing
GO:0007076~mitotic chromosome condensation
GO:0042274~ribosomal small subunit biogenesis
GO:0006397~mRNA processing
GO:0000387~spliceosomal snRNP assembly
GO:0006310~DNA recombination
GO:0051225~spindle assembly
GO:0007051~spindle organization
-log10[Pvalue]
i
Gene ontology analysis of up-regulated genes by p57 overexpression
GO:0006629~lipid metabolic process
GO:0007156~homophilic cell adhesion via plasma membrane adhesion molecules
GO:0007155~cell adhesion
GO:0016477~cell migration
GO:0006695~cholesterol biosynthetic process
GO:0016126~sterol biosynthetic process
GO:0007420~brain development
GO:0008203~cholesterol metabolic process
GO:0007399~nervous system development
GO:0016337~single organismal cell-cell adhesion
GO:0008152~metabolic process
GO:0055114~oxidation-reduction process
GO:0008654~phospholipid biosynthetic process
GO:0006631~fatty acid metabolic process
GO:0007219~Notch signaling pathway
GO:0006694~steroid biosynthetic process
GO:0014068~positive regulation of phosphatidylinositol 3-kinase signaling
GO:0001666~response to hypoxia
GO:0001934~positive regulation of protein phosphorylation
GO:0008284~positive regulation of cell proliferation
-log10[Pvalue]

Supplementary Fig. 1 Overexpression of p57 maintains the undifferentiated state of embryonic cortical NPCs and changes gene expression profile. a Immunohistochemical analysis with antibodies to GFP and to Sox2 for neocortical sections prepared at E17.5 after in utero electroporation (IUE) at E14.5 with an expression plasmid encoding GFP either alone (control) or together with wild-type (WT) or ΔCKI mutant forms of mouse p57. Nuclei were stained with Hoechst 33342. Scale bar, 20 μm. Dashed lines indicate the ventricular zone and the pial surface. **b** Quantification of the proportion of $GFP^{+}Sox2^{+}$ cells among all GFP^{+} cells in the VZ. Five brain sections were analyzed per embryo. Data are means ± SEM (n = 4 or 5 embryos), one-way ANOVA followed by Scheffe's multiple comparison test. **c** Representative flow cytometry for sorting control or p57-expressing NPCs ($GFP^{+}CD133^{+}CD24^{-}$). **d** Mean-average (MA) plots of expression changes in control NPCs and p57-expressing NPCs. Differentially expressed genes (edgeR; $p < 0.05$) are shown in red plots. **e-g** GSEA of the adult quiescent NSCs (**e**), activated NSCs (**f**) and transit amplifying cells (**g**) in SVZ signature genes (Llorens-Bobadilla et al., 2015) in control embryonic NPCs versus p57-expressing embryonic NPCs. NES, normalized enrichment score; FDR, false discovery rate. **h** Gene ontology analysis of biological processes for control NPC-enriched genes (edgeR; $p < 0.03$, expression top 800). **i** Gene ontology analysis of biological processes for p57-expressing NPC-enriched genes (edgeR; $p < 0.03$, expression top 1000).

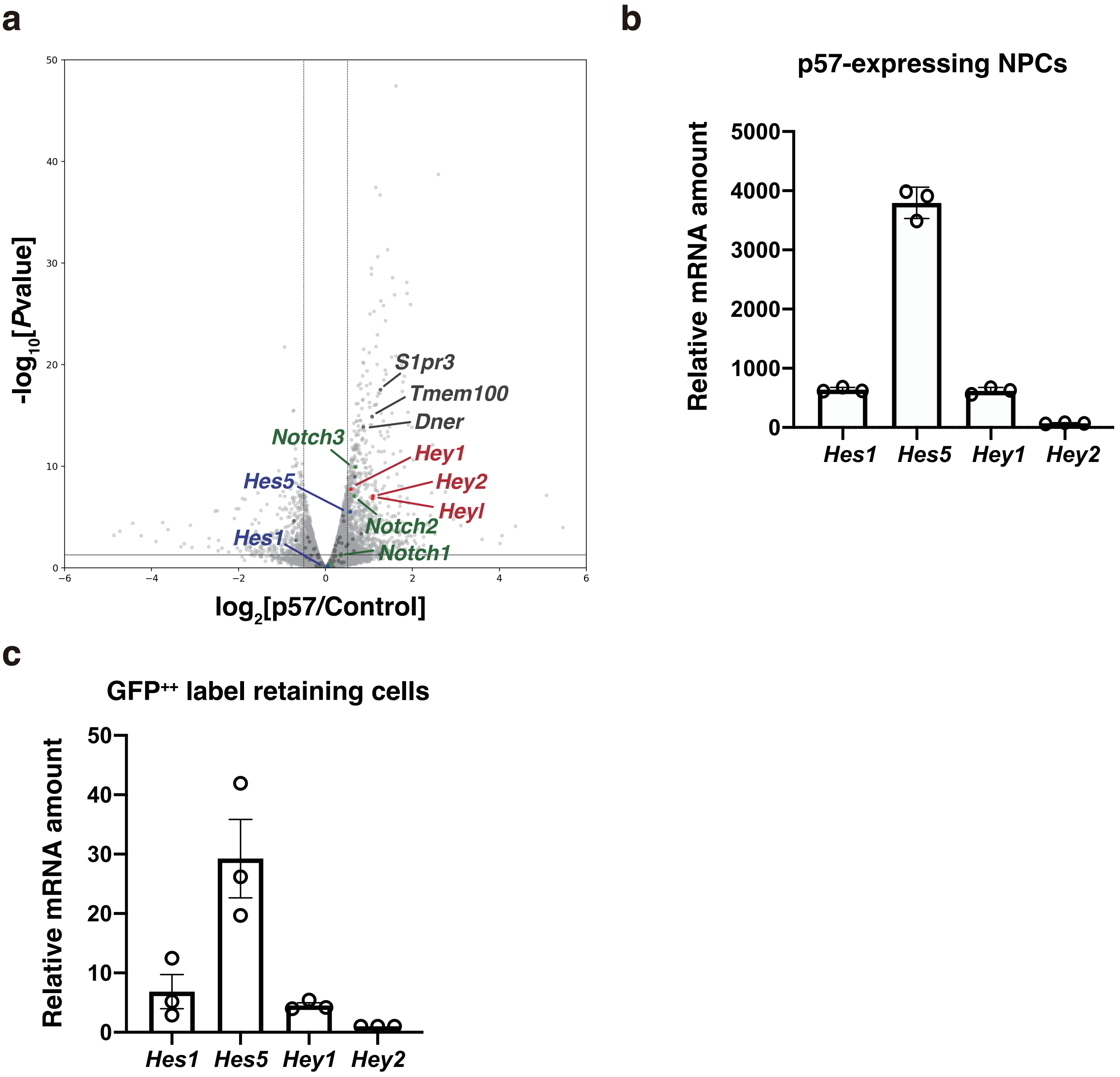

Supplementary Fig. 2 Notch-related genes are differentially expressed between control and p57-expressing NPCs. a Volcano plots showing differentially-expressed genes between control and p57-expressing NPCs. Notch signaling pathway-related genes annotated by the MGI Gene Ontology Browser are highlighted (red, members of the Hey family; blue, members of the Hes family; green, Notch receptors; gray, membrane proteins related to Notch activation). **b** Expression values of the indicated genes from RNA sequencing of NPCs isolated from the neocortices of E17.5 embryos that had been subjected to in utero electroporation at E14.5 with plasmids for GFP and p57 as in Supplementary Fig. 2a. Data are means ± SEM (n = 3 independent experiments). **c** Relative expression values ($2^{-\Delta\Delta Ct}$) of the indicated genes normalized by β-actin Ct value from RT and real-time PCR analysis as in Fig. 3b.

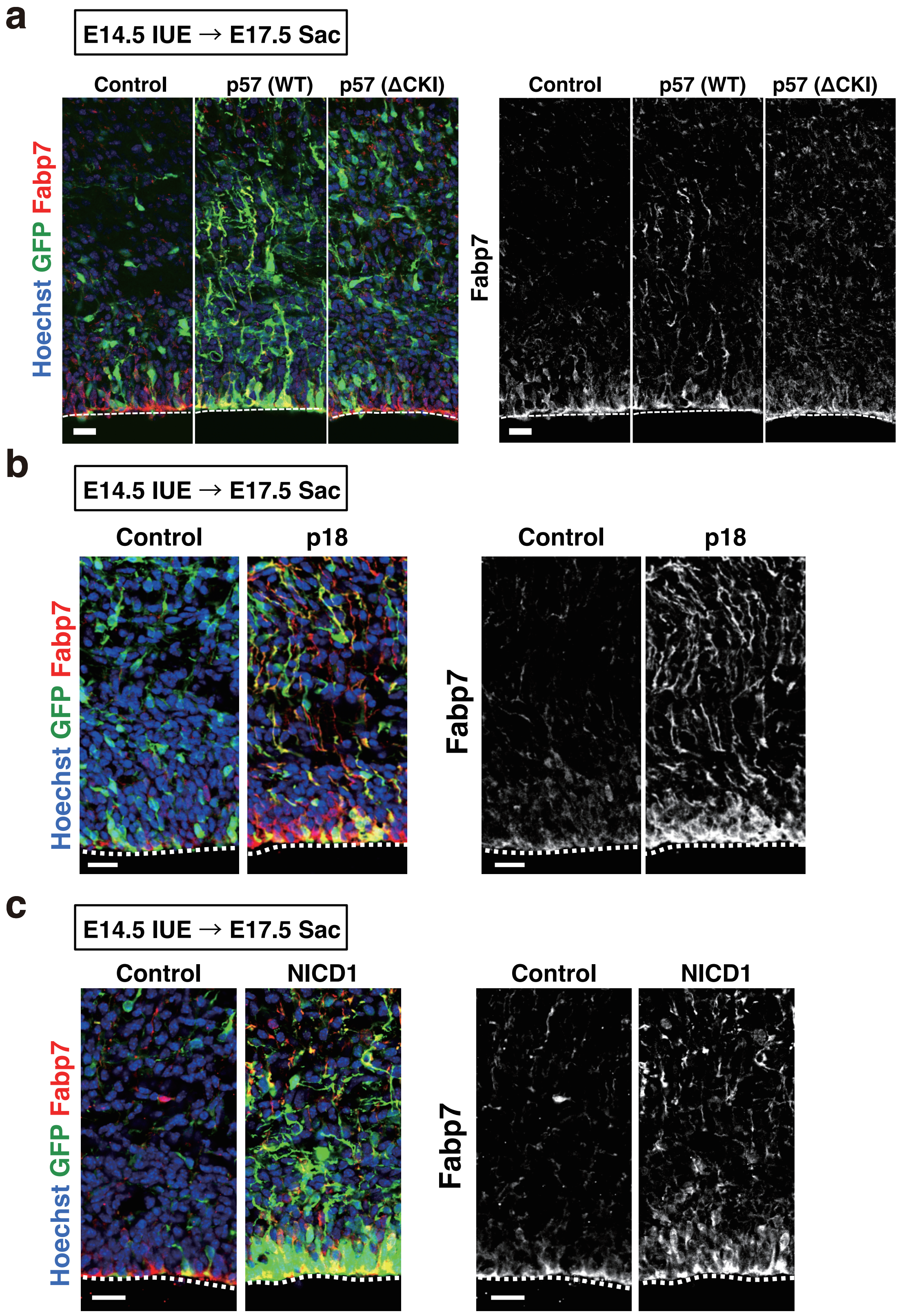

Supplementary Fig. 3 Overexpression of CKIs induces formation of elaborate radial fibers in embryonic cortical NPCs. a-c Neocortical sections prepared at E17.5 after in vitro electroporation at E14.5 with an expression plasmid encoding GFP either alone (control) or together with WT or ΔCKI mutant forms of mouse p57 (**a**), mouse p18 (**b**), or mouse NICD1 (**c**) were subjected to immunohistochemical analysis with antibodies to GFP and to Fabp7. Nuclei were stained with Hoechst 33342. Representative images from n=3 embryos are shown. Scale bars, 20 μm. Dashed lines indicate the ventricular surface.

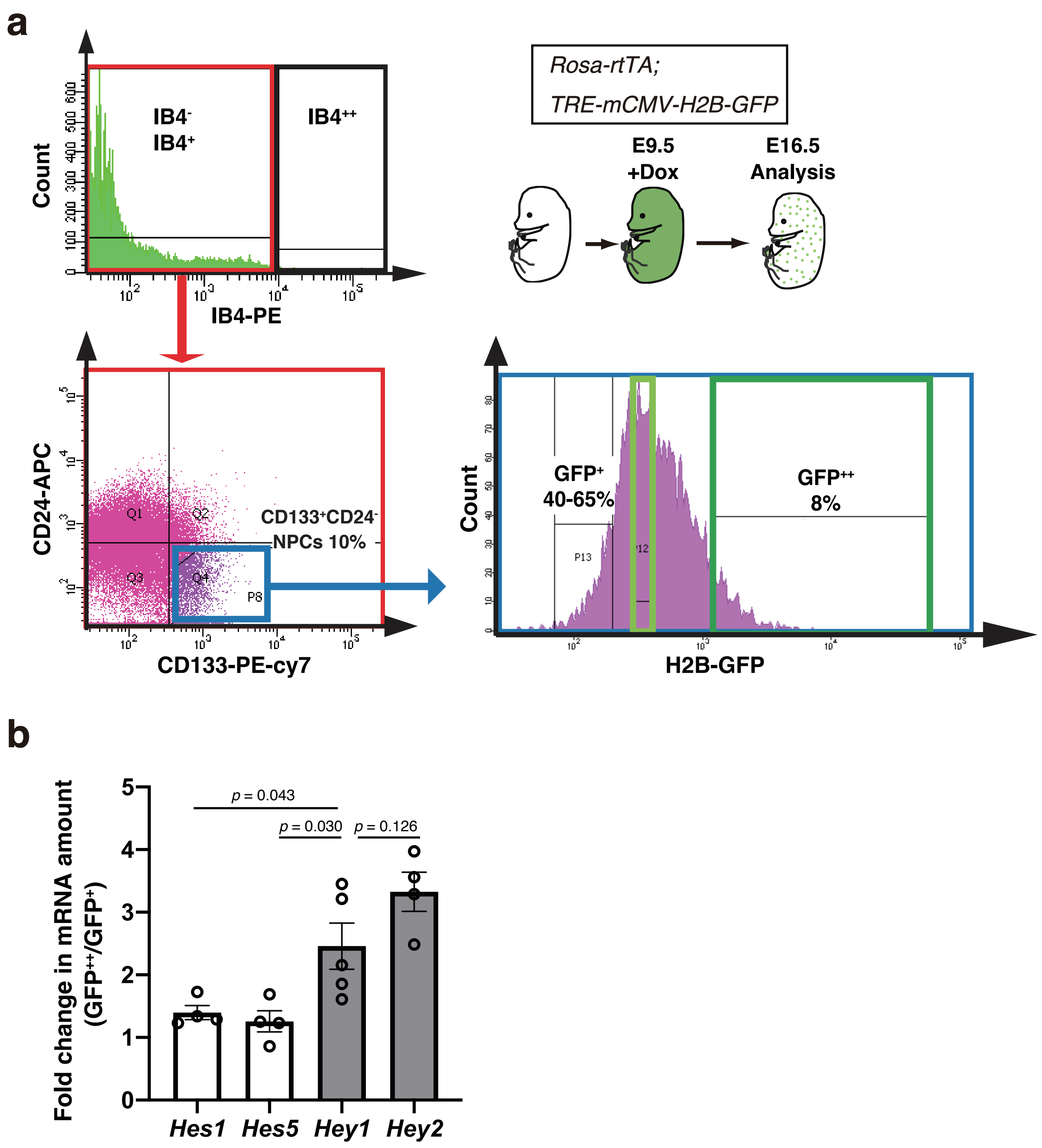

Supplementary Fig. 4 *Hey1* and *Hey2* are highly expressed in slowly dividing NPCs

a Representative flow cytometry for sorting slowly dividing NPCs (GFP^{++}) and rapidly dividing NPCs (GFP^{+}). The top 8% of cells according to H2B-GFP fluorescence intensity (GFP^{++}) were collected as slowly dividing NPCs, whereas the middle 40% to 65% (GFP+ cells) of cells were collected as rapidly dividing NPC fractions. **b** The fold difference in the expression of Notch target genes in GFP^{++} cells relative to that in GFP^{+} cells was determined for the experiment in Fig. 3b. Data are means ± SEM (n = 4, 4, 5, and 4 independent experiments for *Hes1*, *Hes5*, *Hey1*, and *Hey2*, respectively), two-tailed Student's *t* test.

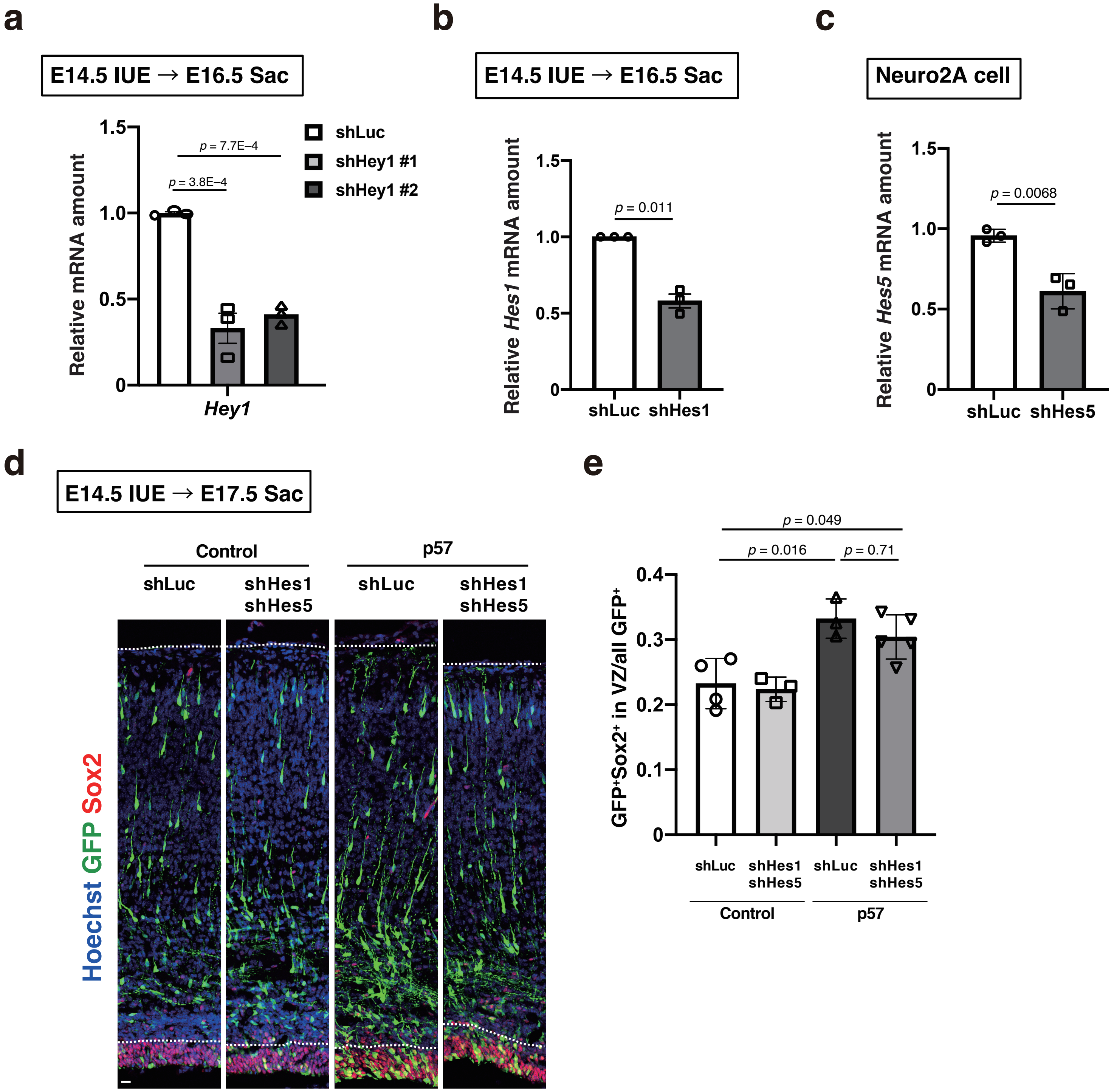
a
E14.5 IUE → E16.5 Sac
Relative mRNA amount
p = 7.7E–4
p = 3.8E–4
shLuc
shHey1 #1
shHey1 #2
Hey1
b
E14.5 IUE → E16.5 Sac
Relative Hes1 mRNA amount
p = 0.011
shLuc
shHes1
c
Neuro2A cell
Relative Hes5 mRNA amount
p = 0.0068
shLuc
shHes5
d
E14.5 IUE → E17.5 Sac
Control
p57
shLuc
shHes1 shHes5
Hoechst GFP Sox2
e
GFP+Sox2+ in VZ/all GFP+
p = 0.049
p = 0.016
p = 0.71
shLuc
shHes1 shHes5
Control
p57

Supplementary Fig. 5 Knockdown efficiency for Hey1, Hes1 and Hes5 shRNAs a The neocortex of mouse embryos subjected to in utero electroporation at E14.5 with vectors for GFP and either control (shLuc) or Hey1 (#1 or #2) shRNAs was dissected at E16.5 for isolation by FACS of cells positive for CD133 and GFP and negative for CD24. The cells were then subjected to RT and real-time PCR analysis of *Hey1* mRNA. Data were normalized by the amount of β-actin mRNA, are expressed relative to the corresponding value for shLuc, and are means ± SEM (n = 3 independent experiments), one-way ANOVA followed by Scheffe's multiple comparison test. **b** The neocortex of mouse embryos subjected to in utero electroporation at E14.5 with vectors for GFP and either control (shLuc) or Hes1 shRNAs was dissected at E16.5 for isolation by FACS of cells positive for CD133 and GFP and negative for CD24. The cells were then subjected to RT and real-time PCR analysis of *Hes1* mRNA. Data were normalized by the amount of β-actin mRNA, are expressed relative to the corresponding value for shLuc, and are means ± SEM (n = 3 independent experiments), two-tailed Student's *t* test. **c** RT and real-time PCR analysis of the abundance of *Hes5* mRNA normalized by that of *GAPDH* mRNA in Neuro2a cells at 1 day after transfection with a vector for Hes5 shRNA or for control shRNA (shLuc). Data are means ± SEM for three independent experiments, two-tailed Student's *t* test. **d** Immunohistochemical analysis with antibodies to GFP and to Sox2 for neocortical sections prepared at E17.5 from embryos that had been subjected to *in utero* electroporation at E14.5 with plasmids for control (shLuc) or Hes1/Hes5 shRNAs and either GFP plus p57 or GFP alone. Nuclei were stained with Hoechst 33342. Scale bars, 20 µm. Dashed lines indicate the ventricular zone and the pial surface. **e** Quantification of the proportion of GFP^+Sox2^+ cells among all GFP^+ cells for sections as in (**d**). Four brain sections were analyzed per embryo. Data are means ± SEM for n = 4, 3, 3 and 5 embryos for shLuc;Control, shHes1/Hes5;Control, shLuc;p57, and shHes1/Hes5;p57, respectively, one-way ANOVA followed by Scheffe's multiple comparison test.

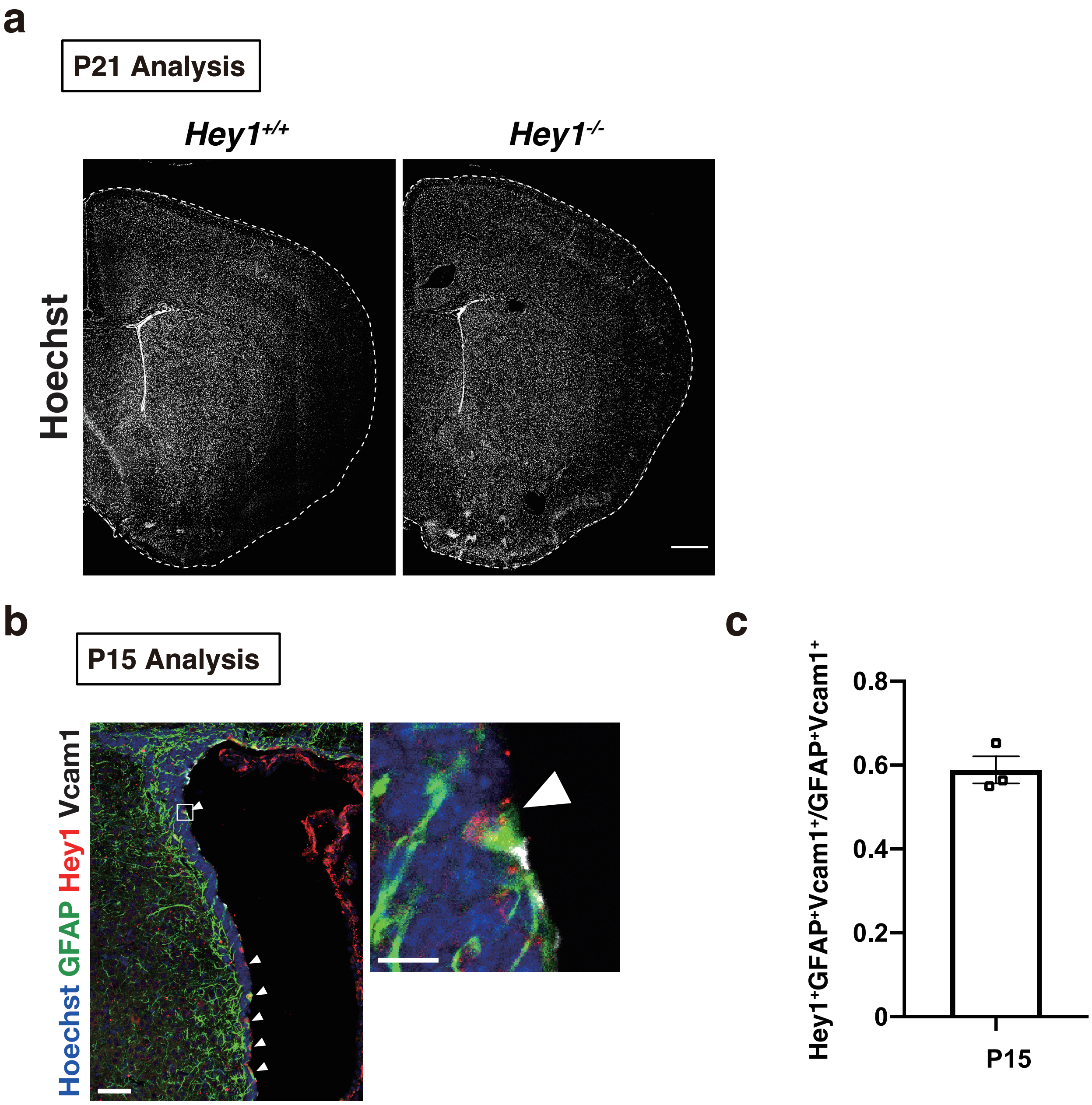

Supplementary Fig. 6 Brain size of Hey1 knockout mice and postnatal Hey1 expression. **a** Fluorescence microscopy of brain sections prepared from Hes1 knockout and control mice at P21 and stained with Hoechst 33342. Representative images from n=3 samples of each genotype are shown. Scale bar, 500 µm. Dashed lines indicate the brain surface. **b** Immunohistochemical analysis with antibodies to GFAP, to Hey1 and to Vcam1 for sections of the ICR mouse brain at postnatal day (P) 15. Nuclei were stained with Hoechst 33342. Arrowheads indicate $GFAP^{+}Vcam1^{+}$ neural stem cells. An image of higher magnification of the boxed region in the left panel is shown as the right panel. Scale bars, 50 µm (left) and 10 µm (right). **c** Quantification of the proportion of $GFAP^{+}Vcam1^{+}Hey1^{+}$ cells among all $GFAP^{+}$ $Vcam1^{+}$ neural stem cells in the SVZ. Four brain sections were analyzed per sample. Data are means ± SEM (n = 3).

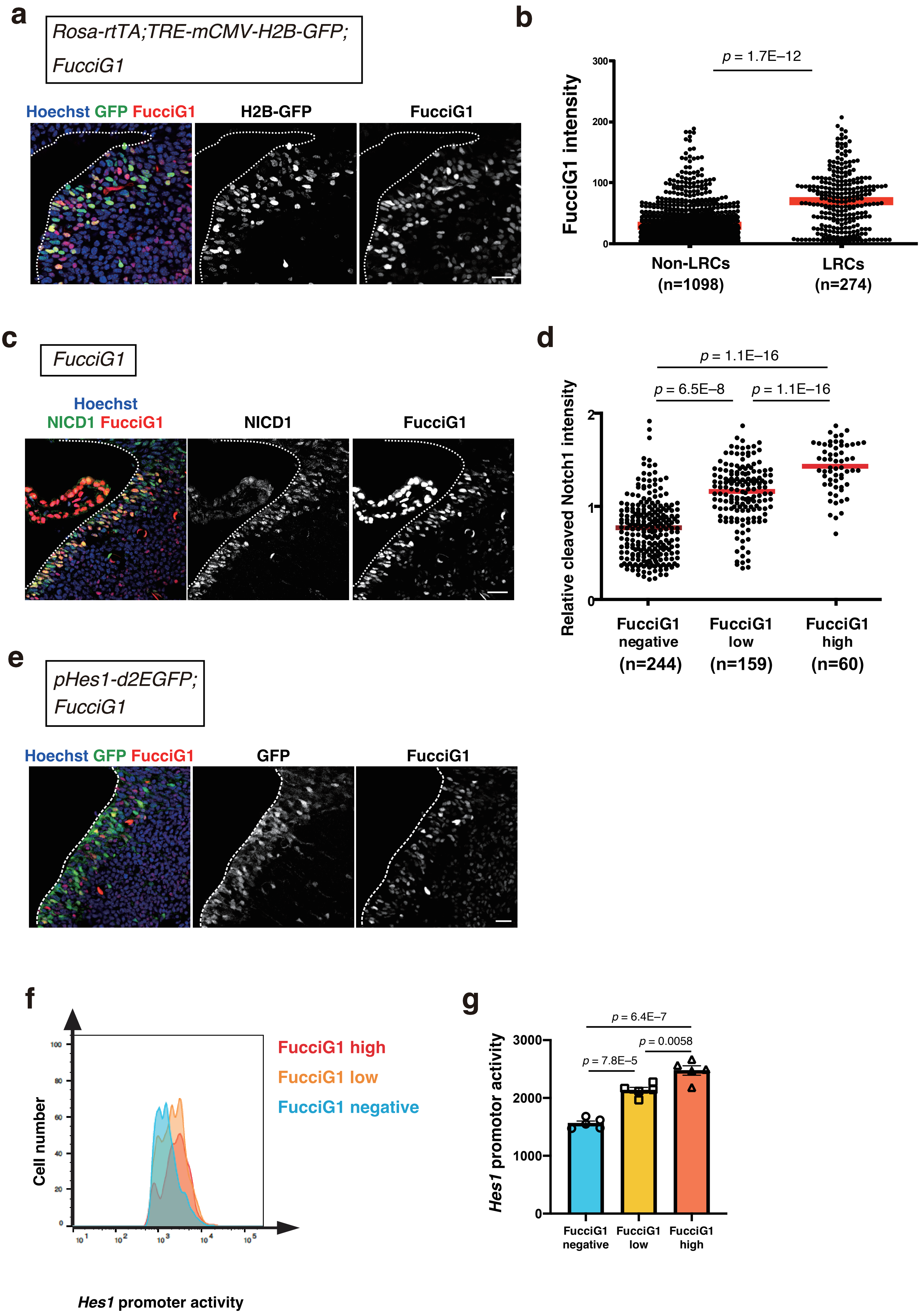
a
Rosa-rtTA;TRE-mCMV-H2B-GFP;
FucciG1
Hoechst GFP FucciG1
H2B-GFP
FucciG1
b
p = 1.7E–12
FucciG1 intensity
300
200
100
0
Non-LRCs
(n=1098)
LRCs
(n=274)
c
FucciG1
Hoechst
NICD1 FucciG1
NICD1
FucciG1
d
p = 1.1E–16
p = 6.5E–8
p = 1.1E–16
Relative cleaved Notch1 intensity
2
1
0
FucciG1
negative
(n=244)
FucciG1
low
(n=159)
FucciG1
high
(n=60)
e
pHes1-d2EGFP;
FucciG1
Hoechst GFP FucciG1
GFP
FucciG1
f
Cell number
100
80
60
40
20
0
FucciG1 high
FucciG1 low
FucciG1 negative
Hes1 promoter activity
g
p = 6.4E–7
p = 0.0058
p = 7.8E–5
Hes1 promotor activity
3000
2000
1000
0
FucciG1
negative
FucciG1
low
FucciG1
high

Supplementary Fig. 7 Most slowly dividing NPCs are in late G_1 phase of the cell cycle. a Immunohistochemical analysis with antibodies to GFP for brain sections of *Rosa-rtTA;TRE-mCMV-H2B-GFP;FucciG1* mice at E17.5 after injection of 9TB-Dox at E9.5. Nuclei were stained with Hoechst 33342. Scale bar, 20 µm. Dashed lines indicate the ventricular surface. **b** Quantification of FucciG1 signals in label-retaining cells (LRCs) and non-LRCs (top 15% and bottom 60% of NPCs for H2B-GFP staining intensity, respectively) in the LGE at E17.5 determined from sections as in (**a**). Data are for the indicated numbers of cells from three independent experiments, with the median being indicated by the horizontal red line, one-sided Mann-Whitney test. **c** Immunohistochemical analysis with antibodies to NICD1 for brain sections of *FucciG1* mice at E17.5. Nuclei were stained with Hoechst 33342. Scale bar, 20 µm. Dashed lines indicate the ventricular surface. **d** Quantification of NICD1 signals in NPCs highly positive for FucciG1 (top 10% of NPCs for FucciG1 intensity), weakly positive for FucciG1 (top 10% to 40% of NPCs for FucciG1 intensity), or negative for FucciG1 (bottom 60% of NPCs for FucciG1 intensity) in the LGE at E17.5 determined from sections as in (**c**). Data are for the indicated numbers of cells from four independent experiments, one-way ANOVA followed by Scheffe's multiple comparison test. **e** Immunohistofluorescence analysis with antibodies to GFP for brain sections of *pHes1-d2EGFP;FucciG1* mice at E17.5. Nuclei were stained with Hoechst 33342. Representative images from n=3 embryos are shown. Scale bar, 20 µm. Dashed lines indicate the ventricular surface. **f** *Hes1* promoter activity (GFP intensity) for NPCs highly positive, weakly positive, or negative for FucciG1 (top 10%, top 10% to 40%, or bottom 60% of NPCs for FucciG1 intensity, respectively) in the LGE at E17.5 as determined by FACS. **g** Quantification of *Hes1* promoter activity for experiments as in (f). Data are means ± SEM (n = 5 embryos), one-way ANOVA followed by Scheffe's multiple comparison test.

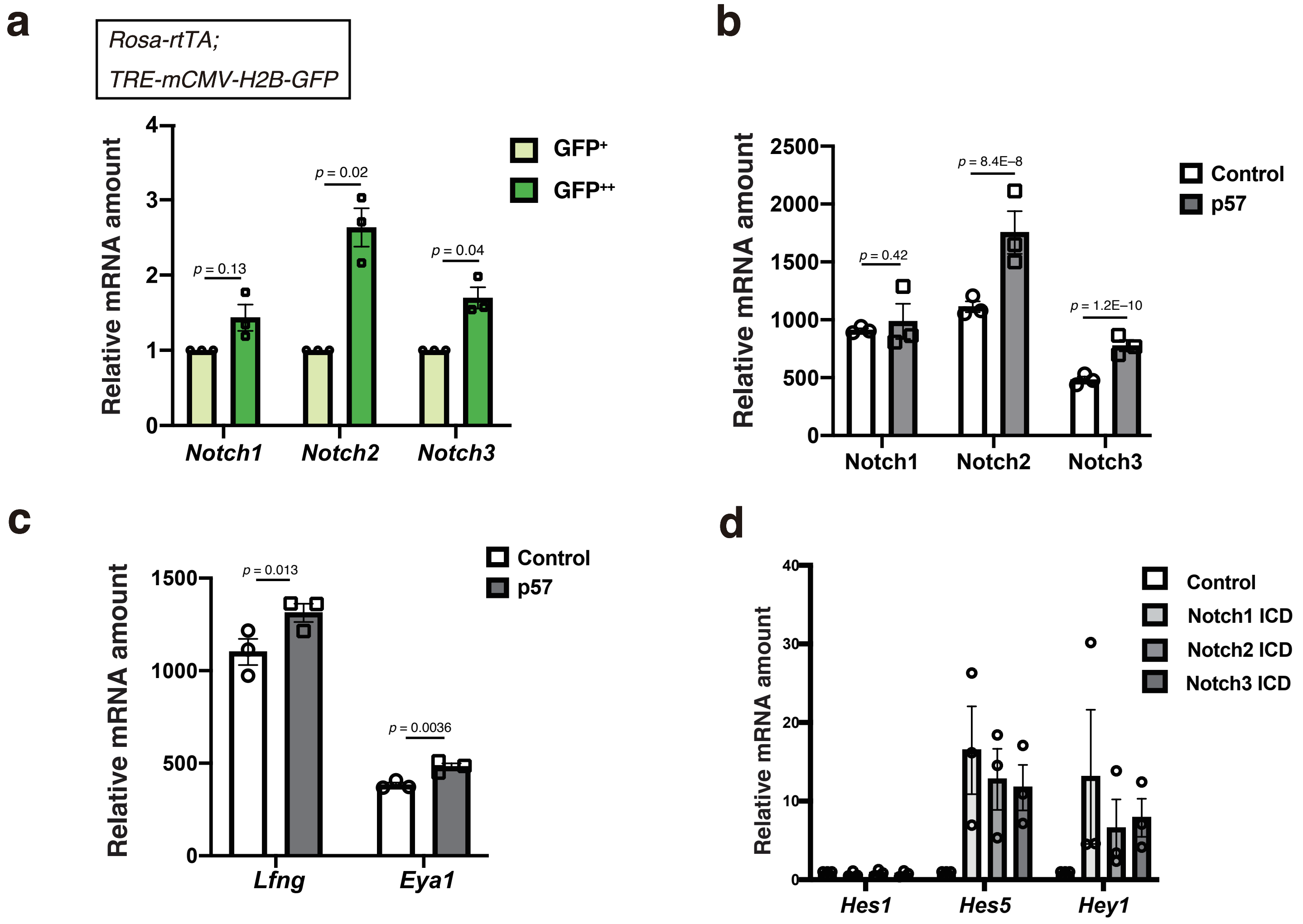

Supplementary Fig. 8 Notch2 and Notch3 are highly expressed in slowly dividing NPCs. a RT and real-time PCR analysis of the indicated genes in NPCs isolated from the LGE of *Rosa-rtTA;TRE-mCMV-H2B-GFP* embryos at E16.5 as in Fig. 3a. Data were normalized by the amount of β-actin mRNA, are expressed relative to the corresponding value for GFP^+ cells, and are means ± SEM (n = 3 independent experiments), two-tailed paired *t* test. **b** Expression values of Notch receptors in NPCs isolated from the neocortex of E17.5 embryos that had been subjected to in utero electroporation at E14.5 with plasmids for GFP alone (control) or together with p57 as in Supplementary Fig. 2a. Data are means ± SEM (n = 3 independent experiments). p values are analyzed by edgeR. **c** Expression values of *Lfng* and *Eya1* in NPCs isolated from the neocortex of E17.5 embryos that had been subjected to in utero electroporation at E14.5 with plasmids for GFP alone (control) or together with p57 as in Supplementary Fig. 2a. Data are means ± SEM (n = 3 independent experiments). p values are analyzed by edgeR. **d** pCAG2-IG (control), pCAG2-NICD1-IG, pCAG2-NICD2-IG or pCAG2-NICD3-IG plasmids were introduced into cultured NPCs by transfection with the use of Lipofectamine 2000. Plasmid-introduced cells were isolated by FACS and then subjected to RT and real-time PCR analysis of the indicated genes. Data were normalized by the amount of β-actin mRNA, are expressed relative to the corresponding value for control, and are means ± SEM (n = 3 independent experiments).

Supplementary Table 1. Quantitative real-time PCR primers

**List of primers for qRT–PCR analysis**		
Gene Name	Forward primer sequence (5' to 3')	Reverse primer sequence (5' to 3')
Actb	5'-AATAGTCATTCCAAGTATCCATGAAA-3'	5'-GCGACCATCCTCCTCTTAG-3'
Gapdh	5'-ATGAATACGGCTACAGCAACAGG-3'	5'-CTCTTGCTCAGTGTCCTTGCTG-3'
Hes1	5'-CACTTCGGACTCCATGT-3'	5'-GAGGTGGGCTAGGGACTTTA-3'
Hes5	5'-AAGTGACTTCTGCGAAGTT-3'	5'-AAGTCCTCTACGGGCTG-3'
Hey1	5'-GTGACCTCGGCCTCCAAA-3'	5'-GTGTCGAAGGGCTCAGTAG-3'
Hey2	5'-CGTTTGCCCATGCAGATT-3'	5'-TGCACAGTGGCCGAAAG-3'
Neurog2	5'-TCGCCAGGGACTGTATCT-3'	5'-CTGTGAAGTGGAGTCCG-3'
Tbr2	5'-CATGGACATCCAGAATGAGC-3'	5'-CAGGAGGAACTAATCTCTTCTTTAAC-3'
Ascl1	5'-CTACGACCCTCTTAGCCC-3'	5'-TCCTGCTTCCAAAGTCCAT-3'
Notch1	5'-GTGGTGCCTCCTAGAGAAA-3'	5'-CCGTCTGTCCTCAGTTG-3'
Notch2	5'-ATTGAACTCTAGGAGGCTTAGGTA-3'	5'-CTTGAGCAAGTTAGCTTATTTCT-3'
Notch3	5'-CCAGGGTGTCTTCCAGATT-3'	5'-CCAGGATCAGTGCAGTAGAG-3'
