# Supplementary material for: Cell cycle arrest determines adult neural stem cell ontogeny by an embryonic Notch-nonoscillatory Hey1 module
Source: Nat Commun. 2021 Nov 12;12:6562. doi: 10.1038/s41467-021-26605-0 (PMC8589987; doi:10.1038/s41467-021-26605-0)
Supplement: Supplementary file 2 — Description of Additional Supplementary Files [file 41467_2021_26605_MOESM2_ESM.pdf]

Description of Additional Supplementary Files

File Name: Supplementary Data 1
Description: **The full list of processed data of RNA sequence of control NPCs and p57-expressing NPCs.** Obtained reads were normalized by TMM normalization as implemented in the R package edgeR. Differential expressed genes were analyzed by edgeR. "Gene ID", "Gene symbol", "Chromosome number", "Start of the read", "End of the read", "Strand", "Length", "Read", "TMM normalized read", "TMM normalized read / length", "a-value", "m-value", "p-value", "q-value", "rank" are provided.

File Name: Supplementary Data 2
Description: **Enriched genes used for GSEA analysis.**
The enriched genes of each cell population used for GSEA analysis are listed in indicated columns.

File Name: Supplementary Movie 1
Description: **The Hes5 promoter confers oscillatory expression dynamics.**
The pHes5-NLS-Ub-Luc2-Hes5-3'UTR plasmid was introduced into cultured NPCs by electroporation together with expression vectors for NICD1 and mCherry. Luciferase levels were visualized by time-lapse bioluminescence imaging over a prolonged period

File Name: Supplementary Movie 2
Description: **The Hey1 promoter confers nonoscillatory expression dynamics.**
The pHey1-NLS-Ub-Luc2-Hey1-3'UTR plasmid was introduced into cultured NPCs by electroporation together with expression vectors for NICD1 and mCherry. Luciferase levels were visualized by time-lapse bioluminescence imaging over a prolonged period.
