# Supplementary material for: Cell cycle arrest determines adult neural stem cell ontogeny by an embryonic Notch-nonoscillatory Hey1 module
Source: Nat Commun. 2021 Nov 12;12:6562. doi: 10.1038/s41467-021-26605-0 (PMC8589987; doi:10.1038/s41467-021-26605-0)
Supplement: Supplementary file 7 — Reporting Summary [file 41467_2021_26605_MOESM7_ESM.pdf]

nature research

Corresponding author(s): Yukiko Gotoh

Last updated by author(s): 2021/09/15

Reporting Summary

Nature Research wishes to improve the reproducibility of the work that we publish. This form provides structure for consistency and transparency in reporting. For further information on Nature Research policies, see our Editorial Policies and the Editorial Policy Checklist.

Statistics

For all statistical analyses, confirm that the following items are present in the figure legend, table legend, main text, or Methods section.

n/a	Confirmed	
☐	☒	The exact sample size (n) for each experimental group/condition, given as a discrete number and unit of measurement
☐	☒	A statement on whether measurements were taken from distinct samples or whether the same sample was measured repeatedly
☐	☒	The statistical test(s) used AND whether they are one- or two-sided<br>*Only common tests should be described solely by name; describe more complex techniques in the Methods section.*
☒	☐	A description of all covariates tested
☐	☒	A description of any assumptions or corrections, such as tests of normality and adjustment for multiple comparisons
☐	☒	A full description of the statistical parameters including central tendency (e.g. means) or other basic estimates (e.g. regression coefficient) AND variation (e.g. standard deviation) or associated estimates of uncertainty (e.g. confidence intervals)
☐	☒	For null hypothesis testing, the test statistic (e.g. F, t, r) with confidence intervals, effect sizes, degrees of freedom and P value noted<br>*Give P values as exact values whenever suitable.*
☒	☐	For Bayesian analysis, information on the choice of priors and Markov chain Monte Carlo settings
☒	☐	For hierarchical and complex designs, identification of the appropriate level for tests and full reporting of outcomes
☒	☐	Estimates of effect sizes (e.g. Cohen's d, Pearson's r), indicating how they were calculated

Our web collection on statistics for biologists contains articles on many of the points above.

Software and code

Policy information about availability of computer code

Data collection	No software was used.
Data analysis	edgeR, Hisat2, GSEA, DAVID

For manuscripts utilizing custom algorithms or software that are central to the research but not yet described in published literature, software must be made available to editors and reviewers. We strongly encourage code deposition in a community repository (e.g. GitHub). See the Nature Research guidelines for submitting code & software for further information.

Data

Policy information about availability of data

All manuscripts must include a data availability statement. This statement should provide the following information, where applicable:

- Accession codes, unique identifiers, or web links for publicly available datasets
- A list of figures that have associated raw data
- A description of any restrictions on data availability

The sequence data have been deposited in the DNA Data Bank of Japan (DDBJ) Sequence Read Archive under the following ID:DRA010678.

Field-specific reporting

Life sciences study design

All studies must disclose on these points even when the disclosure is negative.

Sample size	All data were analyzed with more than three independent experiments.
Data exclusions	No data exclusions.
Replication	All data were replicated more than three times.
Randomization	All sample allocation were randomized.
Blinding	All data were analyzed under blinded conditions.

Reporting for specific materials, systems and methods

We require information from authors about some types of materials, experimental systems and methods used in many studies. Here, indicate whether each material, system or method listed is relevant to your study. If you are not sure if a list item applies to your research, read the appropriate section before selecting a response.

Materials & experimental systems

n/a	Involved in the study	
☐	☒	Antibodies
☒	☐	Eukaryotic cell lines
☒	☐	Palaeontology and archaeology
☐	☒	Animals and other organisms
☒	☐	Human research participants
☒	☐	Clinical data
☒	☐	Dual use research of concern

Methods

n/a	Involved in the study	
☒	☐	ChIP-seq
☐	☒	Flow cytometry
☒	☐	MRI-based neuroimaging

Antibodies

Antibodies used

anti-GFP Abcam Cat# ab13970
anti-GFP Nacalai Tesque Cat# GF090R
anti-Sox2 Cell Signaling Technology Cat# 3728
anti-RFP MBL Cat# PM005
anti-BLBP Millipore Cat# ABN14
anti-S100β Sigma-Aldrich Cat# S2657
anti-S100β Abcam Cat# ab52642
anti-cleaved Notch1 (NICD1) Cell Signaling Technology Cat# 4147
anti-GFAP Abcam Cat# ab4674
anti-Hey1 Millipore Cat# AB5714
anti-Ascl1 BD Biosciences Cat# 556604
anti-PCNA Millipore Cat# NA03
anti-DCX Abcam Cat# ab18723
anti-EGFR Fitzgerald Cat# 20-ES04
Donkey anti-Rabbit IgG (H+L) Highly Cross-Adsorbed Secondary Antibody, Alexa Fluor 488 Thermo Fisher Scientific Cat# A-21206
Donkey anti-Rat IgG (H+L) Highly Cross-Adsorbed Secondary Antibody, Alexa Fluor 488 Thermo Fisher Scientific Cat# A-21208
Donkey anti-Mouse IgG (H+L) Highly Cross-Adsorbed Secondary Antibody, Alexa Fluor 555 Thermo Fisher Scientific Cat# A-31570
Donkey anti-Rabbit IgG (H+L) Highly Cross-Adsorbed Secondary Antibody, Alexa Fluor 555 Thermo Fisher Scientific Cat# A-31572
Donkey anti-Sheep IgG (H+L) Cross-Adsorbed Secondary Antibody, Alexa Fluor 555 Thermo Fisher Scientific Cat# A-21436
Donkey anti-Mouse IgG (H+L) Highly Cross-Adsorbed Secondary Antibody, Alexa Fluor 647 Thermo Fisher Scientific Cat# A-31571
Donkey anti-Rabbit IgG (H+L) Highly Cross-Adsorbed Secondary Antibody, Alexa Fluor 647 Thermo Fisher Scientific Cat# A-31573
Alexa Fluor 488 AffiniPure Donkey Anti-Chicken IgY (IgG) (H+L) antibody Jackson ImmunoResearch Cat# 703-545-155
PE anti-mouse CD133 antibody BioLegend Cat# 141204
PE/Cy7 anti-mouse CD133 antibody BioLegend Cat# 141210
APC anti-mouse CD24 antibody BioLegend Cat# 101814
isolectin GS-IB4 from Griffonia simplicifolia, Alexa Fluor 647 conjugate Thermo Fisher Scientific Cat# I32450

Validation

https://www.abcam.com/gfp-antibody-ab13970.html
https://www.nacalai.co.jp/ss/Contact/PV-CatalogSrchHP.cfm?SearchWord=Anti-GFP(Rat%20IgG2a)%2C%20Monoclonal(GF090R)%2C%20CC&l=EN
https://www.cellsignal.com/products/primary-antibodies/sox2-c70b1-rabbit-mab-ihc-preferred/3728
https://www.mblintl.com/products/pm005/

https://www.merckmillipore.com/JP/ja/product/Anti-Brain-lipid-binding-protein-Antibody,MM_NF-ABN14?ReferrerURL=https%3A%2F%2Fwww.google.com%2F
https://www.sigmaaldrich.com/catalog/product/sigma/s2657?lang=en&region=US
https://www.abcam.com/s100-beta-antibody-ep1576y-astrocyte-marker-ab52642.html
https://www.cellsignal.com/products/primary-antibodies/cleaved-notch1-val1744-d3b8-rabbit-mab/4147
https://www.abcam.com/gfap-antibody-ab4674.html
https://www.merckmillipore.com/JP/en/product/Anti-Hey-1-HRT1-Antibody,MM_NF-AB5714
https://www.bdbiosciences.com/us/applications/research/stem-cell-research/ectoderm-markers/mouse/purified-mouse-anti-mash1-24b72d111/p/556604
https://www.merckmillipore.com/JP/en/product/Anti-PCNA-Ab-1-Mouse-mAb-PC10,EMD_BIO-NA03?ReferrerURL=https%3A%2F%2Fwww.google.co.jp%2F
https://www.abcam.com/doublecortin-antibody-ab18723.html
https://www.citeab.com/antibodies/13040-20-es04-egfr-antibody
https://www.thermofisher.com/antibody/product/Donkey-anti-Rabbit-IgG-H-L-Highly-Cross-Adsorbed-Secondary-Antibody-Polyclonal/A-21206
https://www.thermofisher.com/antibody/product/Donkey-anti-Rat-IgG-H-L-Highly-Cross-Adsorbed-Secondary-Antibody-Polyclonal/A-21208
https://www.thermofisher.com/antibody/product/Donkey-anti-Mouse-IgG-H-L-Highly-Cross-Adsorbed-Secondary-Antibody-Polyclonal/A-31570
https://www.thermofisher.com/antibody/product/Donkey-anti-Rabbit-IgG-H-L-Highly-Cross-Adsorbed-Secondary-Antibody-Polyclonal/A-31572
https://www.thermofisher.com/antibody/product/Donkey-anti-Sheep-IgG-H-L-Cross-Adsorbed-Secondary-Antibody-Polyclonal/A-21436
https://www.thermofisher.com/antibody/product/Donkey-anti-Mouse-IgG-H-L-Highly-Cross-Adsorbed-Secondary-Antibody-Polyclonal/A-31571
https://www.thermofisher.com/antibody/product/Donkey-anti-Rabbit-IgG-H-L-Highly-Cross-Adsorbed-Secondary-Antibody-Polyclonal/A-31573
https://www.jacksonimmuno.com/catalog/products/703-545-155
https://www.biolegend.com/en-us/products/pe-anti-mouse-cd133-antibody-7066
https://www.biolegend.com/en-us/global-elements/pdf-popup/pe-cy7-anti-mouse-cd133-antibody-10193?filename=PE%2FCy7%20anti-mouse%20CD133%20Antibody.pdf&pdfgen=true
https://www.biolegend.com/en-us/products/apc-anti-mouse-cd24-antibody-2937
https://www.thermofisher.com/order/catalog/product/I32450#/I32450

Animals and other organisms

Policy information about studies involving animals; ARRIVE guidelines recommended for reporting animal research

Laboratory animals	Jcl:ICR (CLEA Japan), Slc:ICR (SLC Japan), C57BL/6J
Wild animals	No study involving wild animals.
Field-collected samples	No study collected from the field.
Ethics oversight	All animals were maintained and studied according to protocols approved by the Animal Care and Use Committee of The University of Tokyo.

Note that full information on the approval of the study protocol must also be provided in the manuscript.

Flow Cytometry

Plots

Confirm that:

- [x] The axis labels state the marker and fluorochrome used (e.g. CD4-FITC).
- [x] The axis scales are clearly visible. Include numbers along axes only for bottom left plot of group (a 'group' is an analysis of identical markers).
- [x] All plots are contour plots with outliers or pseudocolor plots.
- [x] A numerical value for number of cells or percentage (with statistics) is provided.

Methodology

Sample preparation	The lateral ganglionic eminence of Rosa-rtTA;TRE-mCMV-H2B-GFP mice at E16.5 or the neocortex of electroporated embryos at E17.5 was dissected and subjected to enzymatic digestion with a papain-based solution (Sumitomo Bakelite), and the dissociated single cells were isolated and incubated for 20 min at room temperature with PBS containing phycoerythrin- and Cy7-conjugated antibodies to CD133 (1:200 dilution, BioLegend, 141210) and allophycocyanin-conjugated antibodies to CD24 (1:200 dilution, BioLegend, 101814)].
Instrument	FACS Aria instrument (Becton Dickinson)

Software	FlowJo
Cell population abundance	We determined the purity of cell populations by checking expression patterns of marker genes using RT-qPCR and RNA-seq analysis.
Gating strategy	We excluded debris by FSC/SSC plot and doublet drops by FSC-hight/FSC-width and SSC-hight/SSC-width plots. In supplemental figure 1, plasmid induced cells are defined by GFP-positive. Then, neural progenitor cells are defined by CD133-positive CD24-negative. In supplemental figure 3, blood cells are excluded by the higher expression of IB4. Then, neural progenitor cells are defined by CD133-positive CD24-negative. Slowly-dividing cells are defined by GFP top 8% and rapidly-dividing cells are defined by GFP middle 40% to 65%.

☒ Tick this box to confirm that a figure exemplifying the gating strategy is provided in the Supplementary Information.
